# Supplementary material for: Myosin-Va and Dynamic Actin Oppose Microtubules to Drive Long-Range Organelle Transport
Source: Curr Biol. 2014 Aug 4;24(15):1743–50. doi: 10.1016/j.cub.2014.06.019 (PMC4131108; doi:10.1016/j.cub.2014.06.019)
Supplement: Document S1. Supplemental Experimental Procedures and Figures S1–S4 [file mmc1.pdf]

Current Biology, Volume *24*
Supplemental Information

Myosin-Va and Dynamic Actin Oppose Microtubules to Drive Long-Range Organelle Transport

Richard D. Evans, Christopher Robinson, Deborah A. Briggs, David J. Tooth, Jose S. Ramalho, Marta Cantero, Lluis Montoliu, Shyamal Patel, Elena V. Sviderskaya, and Alistair N. Hume

Figure S1a-b

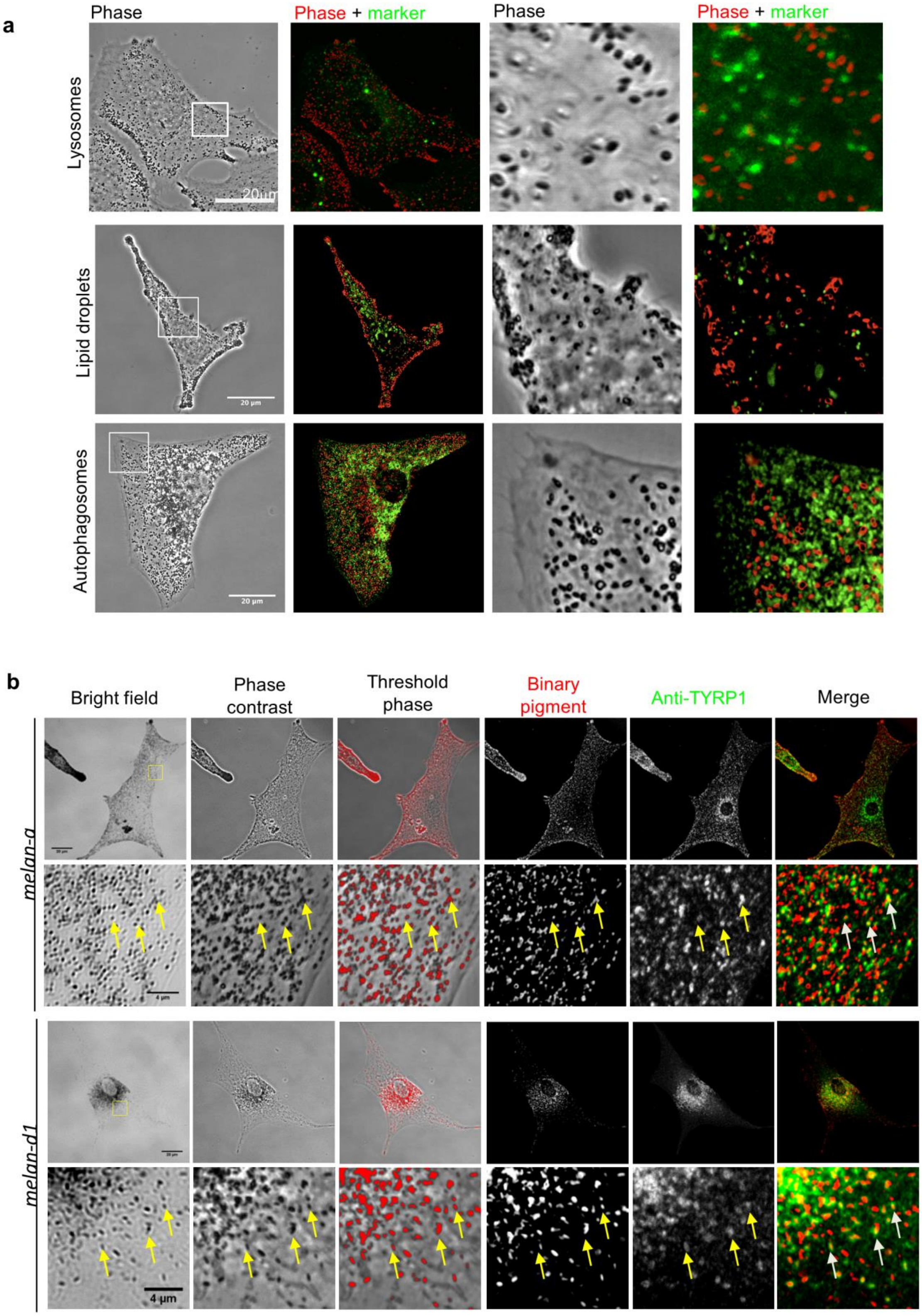
a
Phase
Phase + marker
Phase
Phase + marker
Lysosomes
20μm
Lipid droplets
20 μm
Autophagosomes
20 μm
b
Bright field
Phase contrast
Threshold phase
Binary pigment
Anti-TYRP1
Merge
melan-a
4 μm
melan-d1
4 μm

Figure S1c-e

c

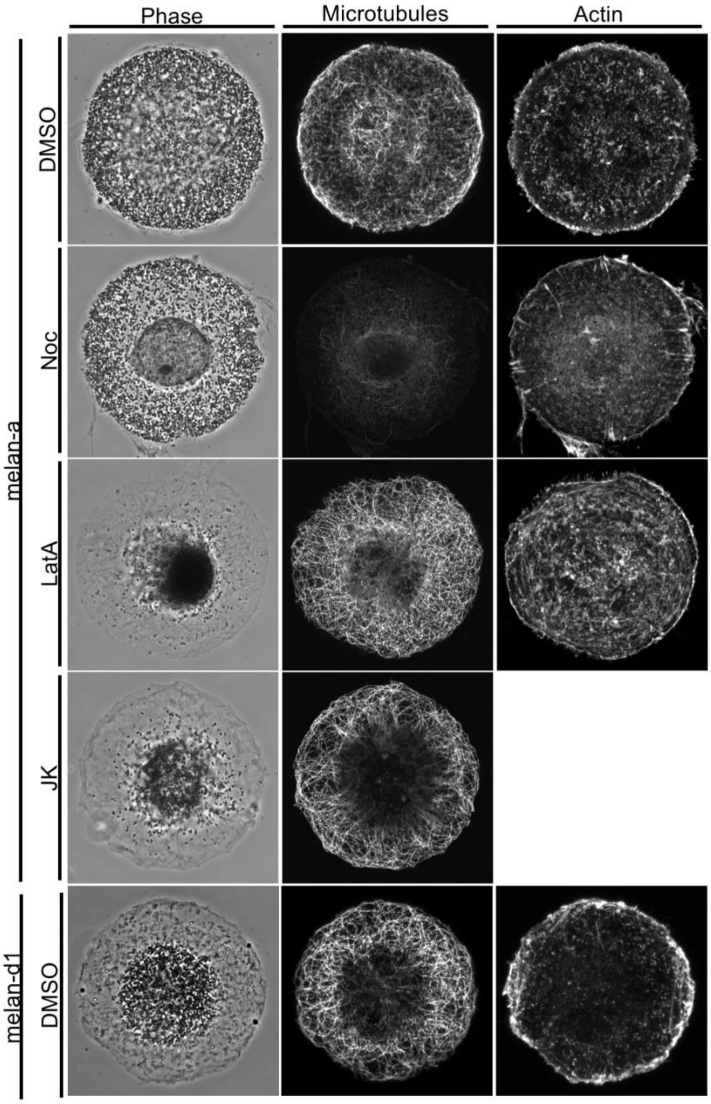

d

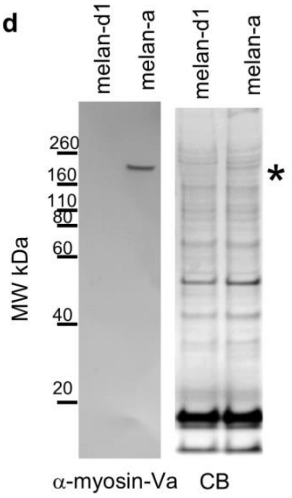

e

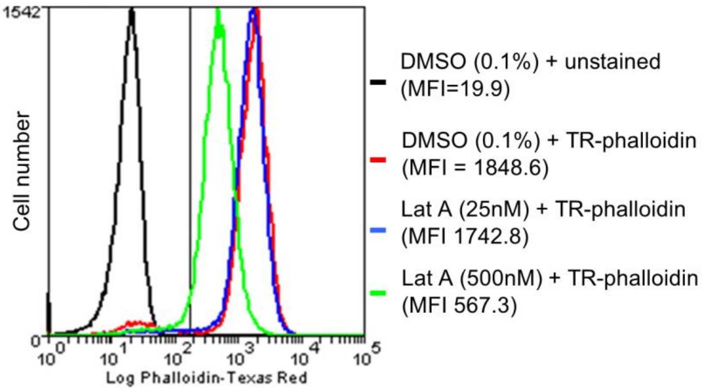

Figure S2

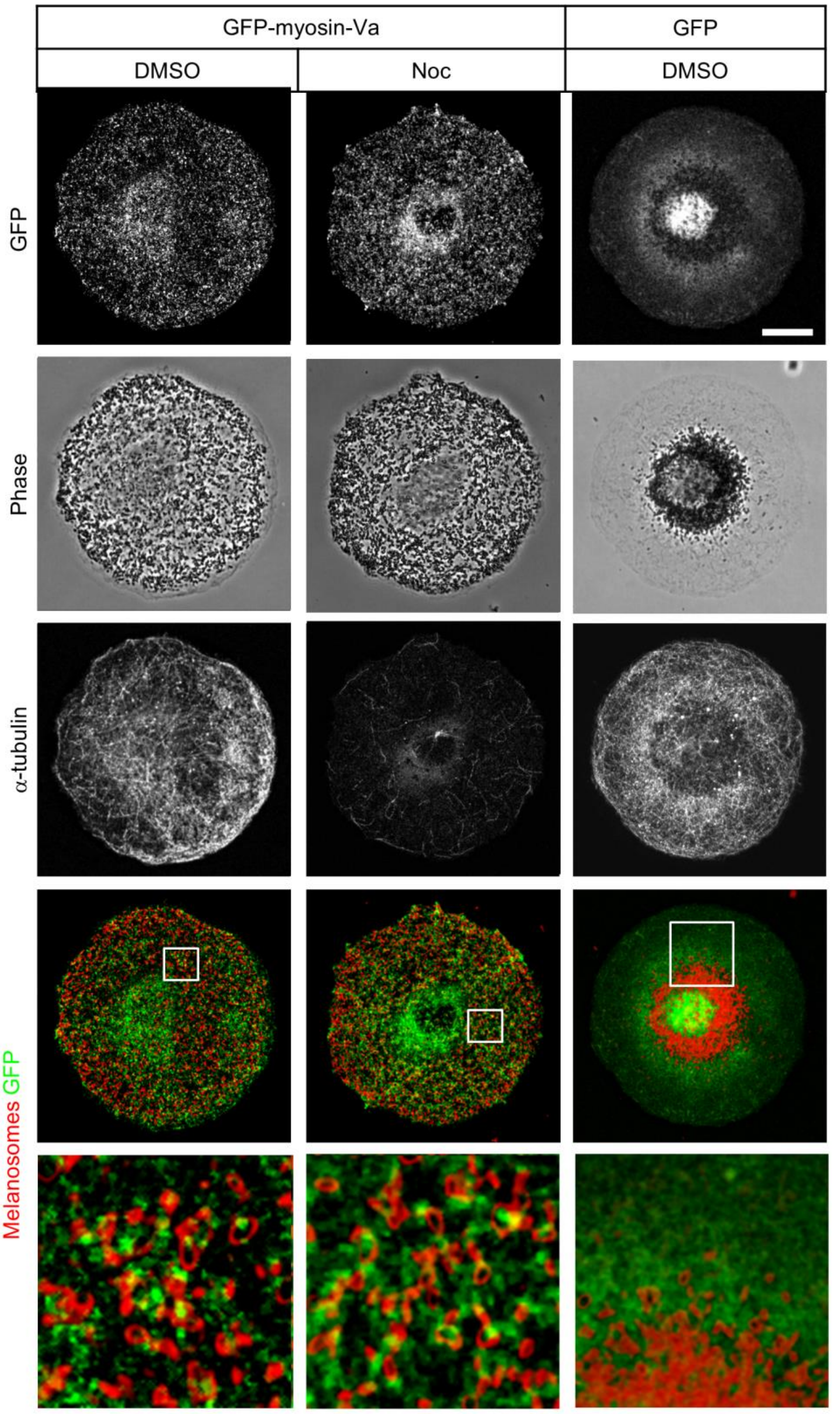
GFP-myosin-Va
GFP
DMSO
Noc
DMSO
GFP
Phase
α-tubulin
Melanosomes GFP

Figure S3a

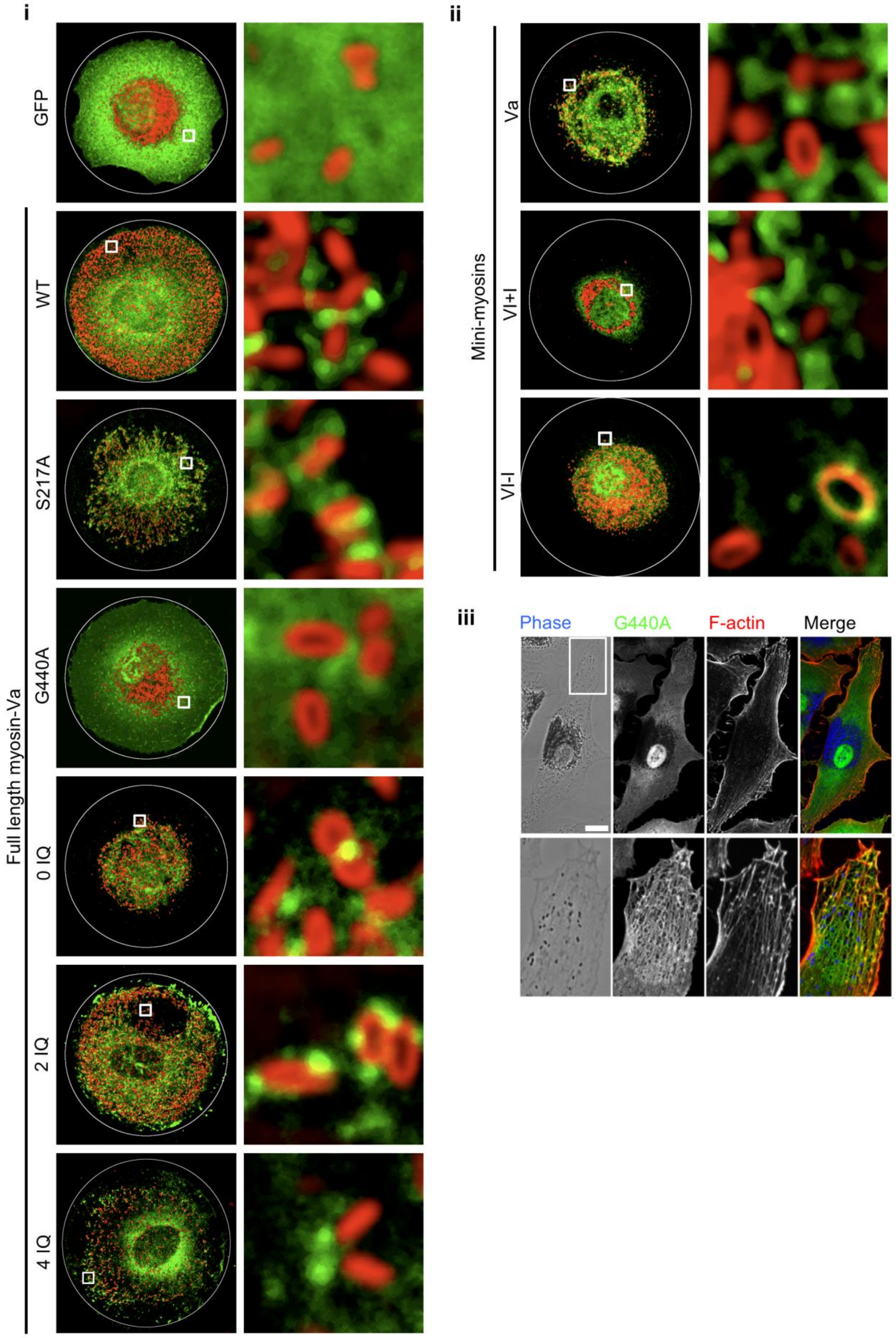
i
GFP
Full length myosin-Va
WT
S217A
G440A
0 IQ
2 IQ
4 IQ
ii
Mini-myosins
Va
VI+I
VI-I
iii
Phase
G440A
F-actin
Merge

Figure S3b

i) melan-ln

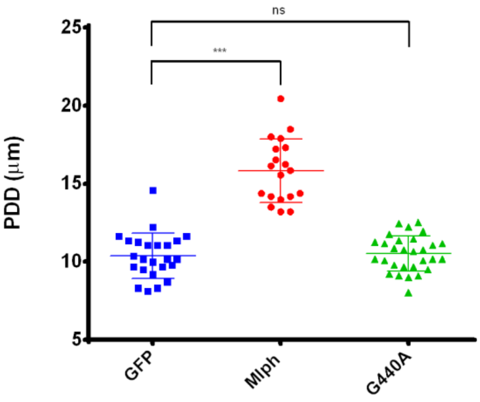

ii) melan-ash

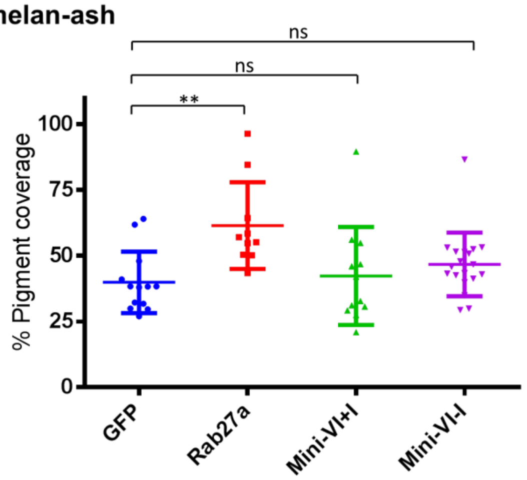

iii) melan-a

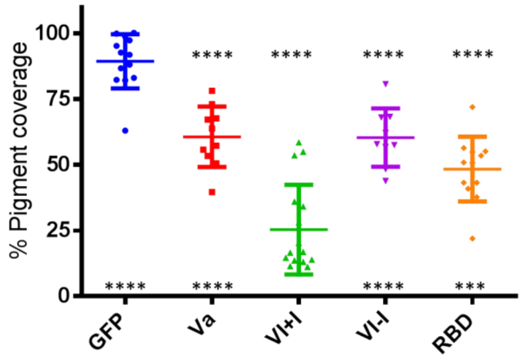

Figure S4

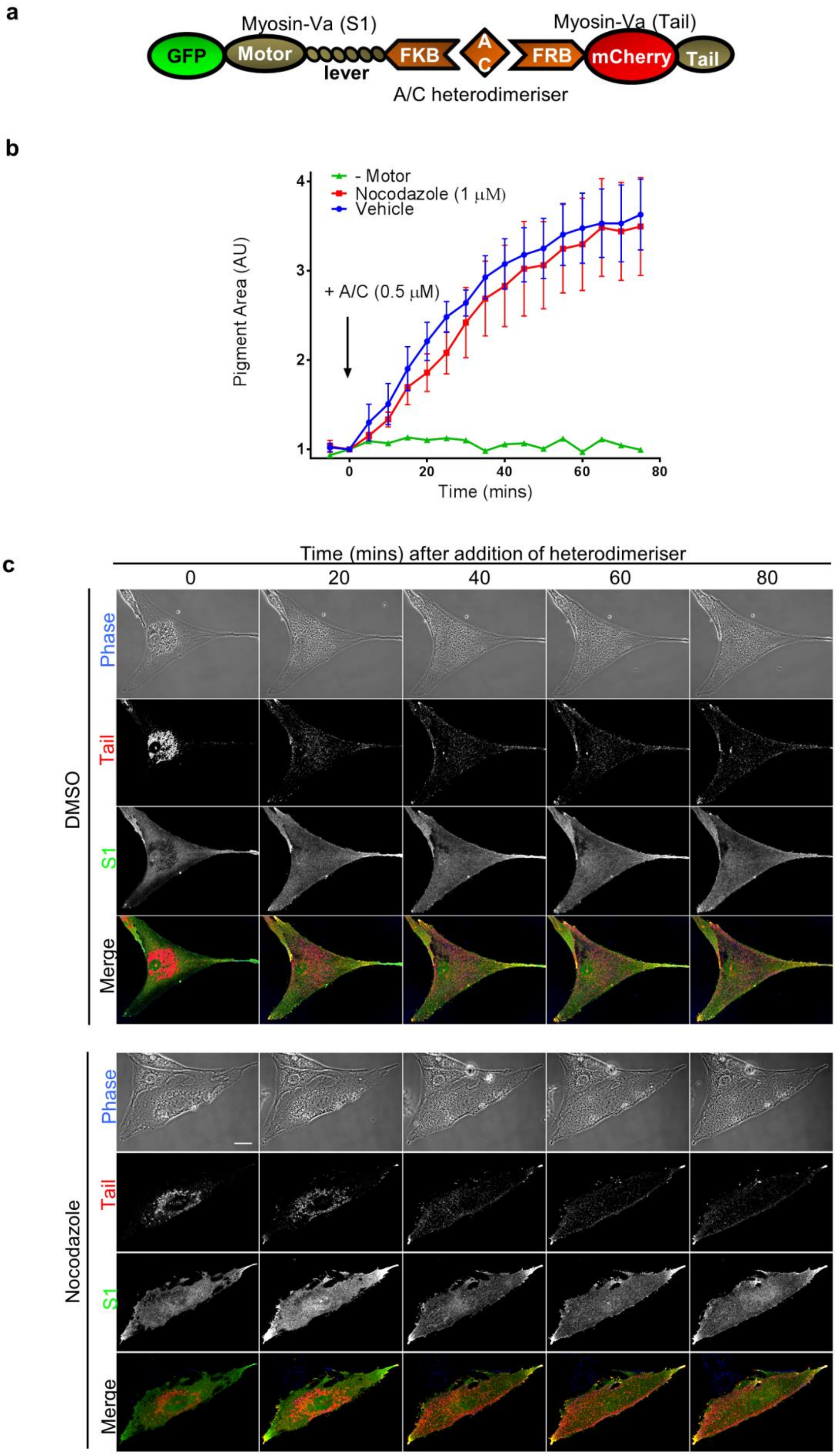
a
Myosin-Va (S1)
GFP
Motor
lever
FKB
A C
FRB
mCherry
Tail
Myosin-Va (Tail)
A/C heterodimeriser
b
- Motor
Nocodazole (1 μM)
Vehicle
Pigment Area (AU)
+ A/C (0.5 μM)
Time (mins)
c
Time (mins) after addition of heterodimeriser
0
20
40
60
80
DMSO
Phase
Tail
S1
Merge
Nocodazole
Phase
Tail
S1
Merge

Supplemental figure legends.

Figure S1 (companion to main figure 1). a) The localisation of highly refractive organelles; lysosomes, lipid droplets and autophagosomes relative to pigmented melanosomes was revealed by lysotracker red, nile red (staining on live cells) and LC3 (staining on chloroquine treated (120mM for 4 hours) fixed cells) staining of melan-a cells. Images are single confocal z-sections at the bottom of the cell where most melanosomes are located. This confirmed that the majority of organelles analysed in this study were melanosomes. b) Tyrosinase-related protein-1 localisation in melan-a and melan-d1 melanocytes, upper and lower rows, respectively. Melanocytes were fixed and stained with rabbit anti-Tyrp-1 (provided by Vince Hearing from the US National Cancer Institute) and then images of melanosomes (bright-field (leftmost column) and phase contrast (second column)) and Tyrp-1 (fifth column) distribution were acquired using a confocal microscope. In the third column we show in red the structures in the phase contrast images (second column) which were included in the binary pigment maps (fourth column). In the rightmost (sixth) column we show the extent of co-localisation between structures in the binary pigment map and Tyrp-1 (highlighted by arrows). Boxes in low magnification images (upper rows) indicate the area presented in the high magnification images (lower rows). Bars = 20μm and 4μm for high and low magnification images, respectively. (c) The distribution of cytoskeleton networks in micro-pattern-grown melanocytes and the effects of cytoskeleton inhibitors. Micro-pattern-grown melan-a and melan-d1 cells were incubated with the indicated cytoskeleton inhibitors or solvent control (0.1% DMSO), fixed and stained with phalloidin and alpha-tubulin-specific antibodies (as described in Experimental procedures). Left-hand, middle and right-hand panels are images showing the distribution of melanosomes (phase contrast), microtubules and actin filaments, respectively. The F-actin distribution in jasplakinolide treated cells is not shown as jasplakinolide binds to F-actin at the same site as phalloidin thus preventing us from visualising F-actin in these cells [1]. Each image = 50 x 50 μm. (d) Confirmation that newly derived immortal dilute lethal 20j (d-l20j) derived melanocytes, melan-d1, lack myosin-Va protein. Lysates of melan-a and melan-d1 cells were immunoblotted with myosin-Va specific antibodies. Commassie blue (CB) staining confirm equal loading of lysate and also highlights a ~200kDa band present in melan-a, but not melan-d1 lysate that may correspond to myosin-Va (asterisk). (e) Flow cytometric analysis of F-actin levels in latrunculin A treated melan-a cells. melan-a cells were incubated for 1 hour with latrunculin A or solvent alone, trypsinised, fixed and stained with texas-red conjugated phalloidin. The fluorescence intensity of 10,000 cells within each population was then measured using a MoFlo fluorescence cytometer (as described in material and methods).

Figure S2 (Companion to main figure 2). Analysis of the intracellular distribution of GFP, microtubules and melanosomes in melan-d1 melanocytes using confocal immunofluorescence microscope. melan-d1 were incubated with 1μM nocodazole or DMSO 0.1% for 1 hour and then infected with adenovirus allowing expression of GFP-myosin-Va or GFP alone in the continued presence of the inhibitor/solvent. Cells were fixed 16 hours later and stained with GFP and α-tubulin specific antibodies (as decribed in Experimental procedures). The white squares in the merged images indicate the part of the cell that is presented in the high magnification panels below. Arrows in high magnification panels indicate examples of colocalisation between melanosomes and GFP-myosin-Va. N.B. in these images some melanosomes appear as hollow red 'O'-shaped structures this is likely to be due to shade-off and halo phase contrast artifacts. High magnification images show

myosin-Va is located adjacent to the highly refractive melanin core of melanosomes, this may reflect differences in z-plane between phase contrast and confocal images. Scale bar = 10μm.

Figure S3 (Companion to main figures 3 + 4). a) Analysis of the subcellular localisation of myosin proteins in melan-d1 melanocytes. i-iii) confocal images showing the intracellular distribution of GFP or the indicated myosin protein (green) and melanosomes (red in i) and ii) and phase/blue in iii)) and F-actin (red in iii)). i) and ii) correspond to proteins tested in main figures 3 and 4, respectively. Left-hand column are over-view images showing entire example cells, while right-hand column are high magnification images (dimensions 2.5 x 2.5μm) showing parts of these cells highlighted in the white boxes shown in the over-views. White circles (left-hand column) indicate the shape of the micropattern (diameter = 46μm). iii) highlights the association of the G440A mutant with F-actin in the peripheral cytoplasm of a free growing cell, lower row are high magnifications images of the boxed region in the upper row. Bar = 20μm. See Fig S2 legend for explanation of the difference in the distribution of GFP and melanin in high magnification images. b) The effect of mini-myosin and control proteins expression upon pigment distribution in melanocytes. i-iii) scatter-plots showing melanosome distribution in micropattern grown (i) or free-growing (ii-iii) melanocytes; melan-ln (Mlph deficient), melan-ash (Rab27a deficient) and melan-a. Melanosome distribution was analysed after either 24 (melan-ash and melan-a) or 48 hours (melan-ln) of plasmid transfection or virus infections, respectively. For ii and iii % pigment coverage (pigment filled area/total cell area) was calculated as described previously [2]. Horzontal bars indicate the median and 25th and 75th percentile of the population in each case. i-ii) The significance of differences in pigment distribution values (PDD or % pigment coverage) between populations are indicated above. iii) The significance of differences in % pigment coverage values for each population compared with the GFP and wild-type myosin-Va are displayed below and above each scatter (**, *** and **** indicate p=<0.01, p=<0.001 and p=<0.0001), respectively.

Figure S4 (companion to main figure 3). Acutely activatable myosin allows direct observation of myosin-Va driven organelle transport in living melanocytes. a) is a schematic summarising the design of the rapamycin (A/C heterodimeriser) activatable myosin-Va (see Supplementary experimental procedures for details). b) is a line-plot showing the change in melanosome distribution, as reported by pigment area, in melan-d1 melanocytes expressing the myosin-Va S1 and tail fragments before and after addition of A/C heterodimeriser (0.5 μM) in the presence and absence of microtubules (n=4 different cells in each case, error bars are standard error of the mean). Pigment area = the x,y area of cytoplasm filled by melanosomes at the indicated time divided by the equivalent area at t=0. c) selected confocal micrographs from one of the time-lapse sequences analysed in b. Upper and lower panel sets show images from sequence recorded from cells in the presence and absence of microtubules, respectively (bar = 20μm).

Experimental procedures.

Plasmid and virus constructs. pENTR529 GFPC2 myosin-Va containing the melanocyte specific exons D and F and lacking brain specific exon B of myosin-Va (XM_006510828.1) was generated by a four-step cloning procedure [3]. Myosin-Va fragments were amplified by RT-PCR from murine melanocytes. pENTR529 was generated from pENTR (Invitrogen) by insertion of EcoRI, NotI, KpnI, SacI and BamHI restriction sites. Step 1: myosin-Va a.a. 1258-1877 coding sequence was subcloned into pENTR529 using internal SacI and BamHI restriction sites to generate pENTR529 myosin-Va 1258-1877. Step 2: myosin-Va a.a 709-1258 coding sequence was subcloned into pENTR529 myosin-Va 1258-1877 using KpnI (internal) and SacI restriction sites to generate pENTR529 myosin-Va 709-1877. Step 3: EGFP coding sequence was PCR amplified from pEGFP-C2 (Clontech) and sub-cloned into pENTR529 myosin-Va 709-1828 using EcoRI/NotI restriction sites to generate pENTR529 GFPC2 myosin-Va 709-1877. Step 4: myosin-Va a.a 1-709 coding sequence subcloned into pENTR529 GFPC2 myosin-Va 709-1877 using NotI and KpnI restriction sites to generate pENTR529 GFPC2 myosin-Va. Myosin-Va mutants; G440A, S217A, 0IQ, 2IQ and 4IQ, (lacking amino acids 762-920, 810-920 and 858-920, respectively)were generated by Quikchange site-directed mutagenesis. Mini-myosin myosin-VI (+/-insert)/Va chimera (mini-VI+I, VI-I and Va) were as described in [4] and from the N-terminus comprised; EGFP fused to the motor domain of human myosin-VI (NM_010864.2) including (aa 1-809; VI+I) or lacking (aa 1-771; VI-I) the unique insert region responsible for the directionality of myosin-VI or murine myosin-Va (aa 1-761; Va), fused to the lever arm of murine myosin Va (aa 762-920) and the Rab27a binding domain of murine Sytl2a (aa 1-90; RBD) (NM_031394). For inducibly active myosin-Va 2 constructs were made; 1) Myosin-Va S1 (from the N-terminus) GFP-motor-lever-FKBP fusion was made by replacement of the RBD of the mini-Va construct with human FK506 binding protein 1A (FKBP) (NM_000801.4) (aa 1-108) (Source Bioscience) and 2) Myosin-Va tail (from the N-terminus) FRB-mCherry-myosin-Va (melanosome/melanophilin binding tail) was made by sequential sub-cloning of human FKBP-rapamycin–binding domain (FRB) of mTOR (mammalian target of Rapamycin; L35478.1) (aa34-127) (Source Bioscience), mCherry and murine myosin-Va (aa 1277-1895) into the pENTR-V5 vector previously described [2]. Primer sequences are available upon request. Adenoviruses allowing expression of the GFP-myosin-Va and mutant variants were generated as previously described [2].

Melanocyte culture. To generate immortal melan-d1 melanocytes C57BL6/J mice heterozygous for the dilute lethal-20J (d-l20J) mutation were crossed with C57BL6/J mice carrying an Ink4a-Arf exon 2 deletion [5]. Trunk skin of a neonatal F2 d-l20J/d-l20J mouse was used for preparation of melanocyte cultures, as described previously [6], yielding immortal line melan-d1. Confocal immunofluorescence microscopy and Western blotting confirmed that these cells contain a normal complement of mature melanosomes and lack expression of myosin-Va (Fig S1b+d). melan-d1 cells were maintained in RPMI 1640 medium supplemented with 10% fetal calf serum, 2 mM glutamine, 100 U/ml penicillin G, and 100 mg/ml streptomycin, 200 nM phorbol 12-myristate 13-acetate, 200 pM cholera toxin (all from Sigma-Aldrich, Poole, United Kingdom), at 37°C with 10% CO_2. Cultures of immortal melan-a, melan-ln and melan-ash cells were maintained as described previously [7]. Cytoskeleton inhibitors were added to full melanocyte growth medium to 1μM nocodazole, 25nM latrunculin-A and 8nM jasplakinolide.

Immunofluorescence microscopy. Cells for immunofluorescence were paraformaldehyde fixed, stained and fluorescence and transmitted light images of melanocytes were then collected using a

Zeiss LSM710 confocal microscope fitted with a 63x 1.4NA oil immersion Apochromat lens. All images presented are single sections in the *z*-plane. Antibodies and stains were used as indicated; mouse monoclonal anti-GFP (Roche 11814460001; 1:200) mouse-anti-α-tubulin (1:100 Sigma clone DM-1) texas-red-phalloidin (Sigma P1951; 100nM), goat anti-mouse Alexa488 labelled secondary antibodies (Molecular Probes A-11001; 1:500). For live cell experiments using activatable myosin-Va, melan-d1 cells were plated 35mm diameter glass bottomed petri dishes (Matek P35G-1.5-20-C) ($1x10^4$ cells/dish) and the next day infected with adenoviruses allowing expression of GFP-myosin-Va(S1)-FKBP and FRB-mCherry-myosin-Va tail. 48 hours later dishes were transferred to the stage of the Zeiss LSM710 confocal microscope within an environmental chamber (37°C), medium was replaced with L-15 medium supplemented with 10% fetal calf serum, 100 U/ml penicillin G, and 100 mg/ml streptomycin and 0.5μM rapamycin analogue (A/C heterodimeriser, Clontech). Images of GFP, mCherry and melanosomes were then acquired at a frame rate of 12/hour.

Immunoblotting. Immunoblotting was performed as described previously [7] using rabbit anti-myosin-Va (Cell Signaling Technology #3402 1:1000).

Flow cytometry. Samples were analysed using a Beckman Coulter MoFlo XDP flow cytometer, equipped with 488nm and 561nm lasers to obtain forward and side scatter and excite Texas-Red respectively. Emitted fluorescence light was collected using 615/20nm band pass filter. Data was analysed using Weasel Software Version 3.0.2 (The Walter and Eliza Hall Institute of Medical Research, Melbourne Australia). Cells were gated based upon based upon their forward and side scatter profile, to exclude debris and overall mean fluorescence intensity (MFI) were reported for each population.

References.

1. Bubb, M.R., Senderowicz, A.M., Sausville, E.A., Duncan, K.L., and Korn, E.D. (1994). Jasplakinolide, a cytotoxic natural product, induces actin polymerization and competitively inhibits the binding of phalloidin to F-actin. The Journal of biological chemistry *269*, 14869-14871.
2. Hume, A.N., Tarafder, A.K., Ramalho, J.S., Sviderskaya, E.V., and Seabra, M.C. (2006). A coiled-coil domain of melanophilin is essential for Myosin Va recruitment and melanosome transport in melanocytes. Molecular biology of the cell *17*, 4720-4735.
3. Seperack, P.K., Mercer, J.A., Strobel, M.C., Copeland, N.G., and Jenkins, N.A. (1995). Retroviral sequences located within an intron of the dilute gene alter dilute expression in a tissue-specific manner. EMBO J *14*, 2326-2332.
4. Park, H., Li, A., Chen, L.Q., Houdusse, A., Selvin, P.R., and Sweeney, H.L. (2007). The unique insert at the end of the myosin VI motor is the sole determinant of directionality. Proc Natl Acad Sci U S A *104*, 778-783.
5. Lavado, A., Matheu, A., Serrano, M., and Montoliu, L. (2005). A strategy to study tyrosinase transgenes in mouse melanocytes. BMC Cell Biol *6*, 18.
6. Sviderskaya, E.V., Hill, S.P., Evans-Whipp, T.J., Chin, L., Orlow, S.J., Easty, D.J., Cheong, S.C., Beach, D., DePinho, R.A., and Bennett, D.C. (2002). p16(Ink4a) in melanocyte senescence and differentiation. J Natl Cancer Inst *94*, 446-454.
7. Hume, A.N., Ushakov, D.S., Tarafder, A.K., Ferenczi, M.A., and Seabra, M.C. (2007). Rab27a and MyoVa are the primary Mlph interactors regulating melanosome transport in melanocytes. J Cell Sci *120*, 3111-3122.
