# Supplementary figures and images for: Myosin-Va and Dynamic Actin Oppose Microtubules to Drive Long-Range Organelle Transport
Source: Curr Biol. 2014 Aug 4;24(15):1743–50. doi: 10.1016/j.cub.2014.06.019 (PMC4131108; doi:10.1016/j.cub.2014.06.019)

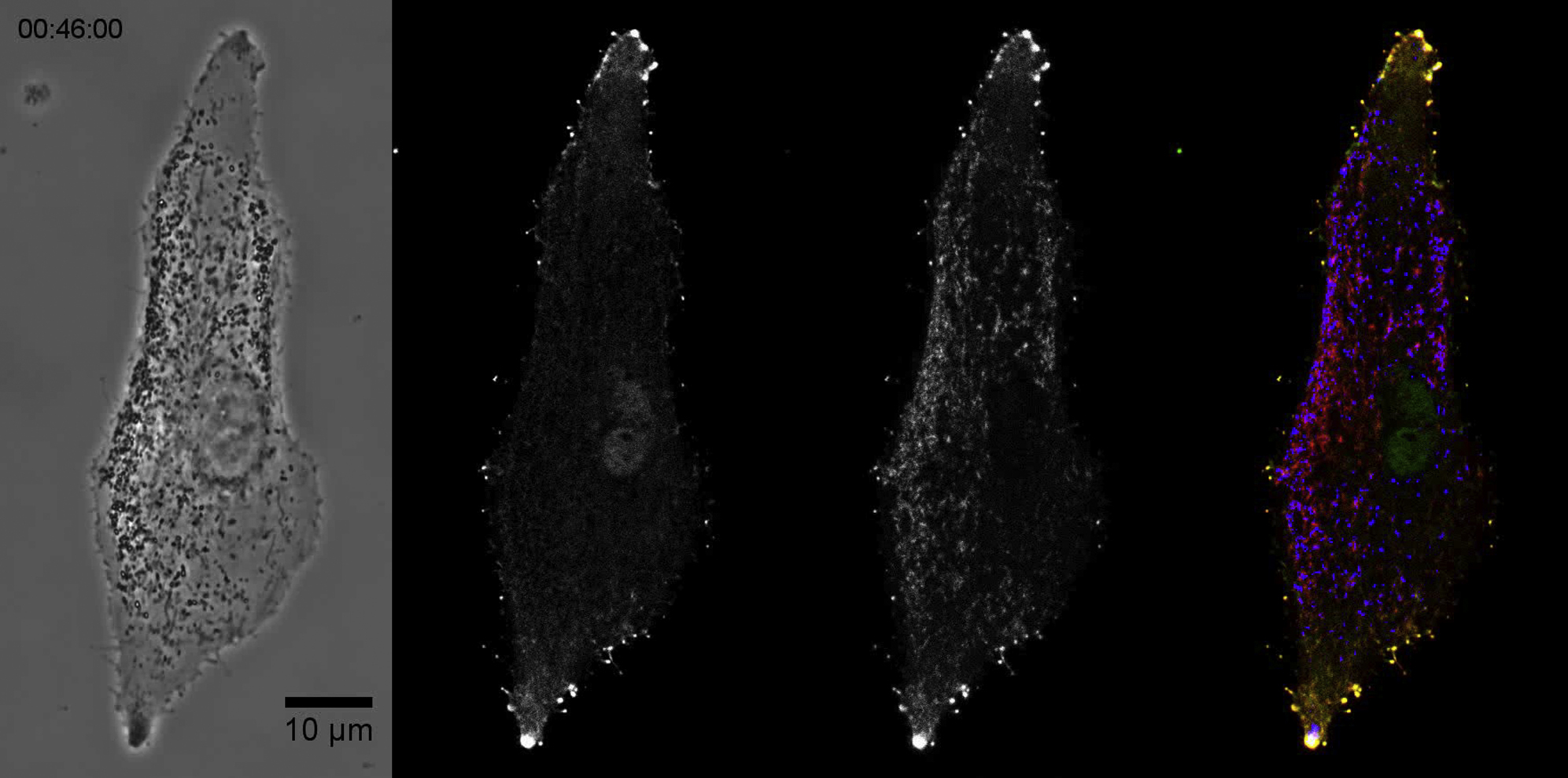

Supplement: Movie S1. Acute Activation of Myosin-Va in Melan-d1 Melanocytes Results in Rapid Redistribution of Melanosomes from the Cell Center to the Periphery without Significant Changes in Cell Shape — From left, images show melanosomes (phase-contrast-inverted binary blue in merge), myosin-Va S1 (green in merge), myosin-Va tail (red in merge), and merge. Frame rate is 5 frames/s (240× real time). [file mmc2.jpg]
