# Supplementary material for: Myosin-Va and Dynamic Actin Oppose Microtubules to Drive Long-Range Organelle Transport
Source: Curr Biol. 2014 Aug 4;24(15):1743–50. doi: 10.1016/j.cub.2014.06.019 (PMC4131108; doi:10.1016/j.cub.2014.06.019)
Supplement: Document S2. Article plus Supplemental Information [file mmc3.pdf]

Current Biology 24, 1743–1750, August 4, 2014 ©2014 The Authors http://dx.doi.org/10.1016/j.cub.2014.06.019

Report

Myosin-Va and Dynamic Actin Oppose Microtubules to Drive Long-Range Organelle Transport

Richard D. Evans,[1,6] Christopher Robinson,[1,6] Deborah A. Briggs,[1] David J. Tooth,[1] Jose S. Ramalho,[2] Marta Cantero,[3,4] Lluis Montoliu,[3,4] Shyamal Patel,[5] Elena V. Sviderskaya,[5] and Alistair N. Hume[1,*]
[1]School of Life Sciences, University of Nottingham, Nottingham NG7 2UH, UK
[2]CEDOC Faculdade de Ciencias Medicas, Universidade Nova de Lisboa, 1169-056 Lisbon, Portugal
[3]Centro Nacional de Biotecnologia (CNB-CSIC), Madrid 28049, Spain
[4]CIBERER-ISCIII, Madrid 28029, Spain
[5]Cell Signalling Research Centre, Division of Biomedical Sciences, St. George's, University of London, London SW17 0RE, UK

Summary

In animal cells, microtubule and actin tracks and their associated motors (dynein, kinesin, and myosin) are thought to regulate long- and short-range transport, respectively [1–8]. Consistent with this, microtubules extend from the perinuclear centrosome to the plasma membrane and allow bidirectional cargo transport over long distances (>1 μm). In contrast, actin often comprises a complex network of short randomly oriented filaments, suggesting that myosin motors move cargo short distances. These observations underpin the "highways and local roads" model for transport along microtubule and actin tracks [2]. The "cooperative capture" model exemplifies this view and suggests that melanosome distribution in melanocyte dendrites is maintained by long-range transport on microtubules followed by actin/myosin-Va-dependent tethering [5, 9]. In this study, we used cell normalization technology to quantitatively examine the contribution of microtubules and actin/myosin-Va to organelle distribution in melanocytes. Surprisingly, our results indicate that microtubules are essential for centripetal, but not centrifugal, transport. Instead, we find that microtubules retard a centrifugal transport process that is dependent on myosin-Va and a population of dynamic F-actin. Functional analysis of mutant proteins indicates that myosin-Va works as a transporter dispersing melanosomes along actin tracks whose +/barbed ends are oriented toward the plasma membrane. Overall, our data highlight the role of myosin-Va and actin in transport, and not tethering, and suggest a new model in which organelle distribution is determined by the balance between microtubule-dependent centripetal and myosin-Va/actin-dependent centrifugal transport. These observations appear to be consistent with evidence coming from other systems showing that actin/myosin networks can drive long-distance organelle transport and positioning [10, 11].

[6]Co-first author
*Correspondence: alistair.hume@nottingham.ac.uk
This is an open access article under the CC BY license (http://creativecommons.org/licenses/by/3.0/).

Results and Discussion

To understand how the microtubule and actin transport systems cooperate to regulate organelle transport, we tested the effect of their depletion on melanosome distribution in wild-type melan-a cells. For this, we incubated cells with either nocodazole or latrunculin A to specifically deplete microtubules or F-actin, respectively. We then used light microscopy to examine the effects of these treatments upon intracellular melanosome distribution. To facilitate the quantitative analysis of melanosome distribution, in these and subsequent experiments, we standardized melanocyte shape in the x and y planes by growing cells on coverslips containing fibronectin micropatterns (see Experimental Procedures). In this condition, melanocytes adopted a uniform circular shape determined by the micropattern, with the nucleus positioned near the center and the melanosomes distributed throughout the surrounding cytoplasm. This circumvented the need for manual measurements (described previously) [12] and allowed for the semiautomated measurement of melanosome distribution in large populations of cells (see Experimental Procedures). We report melanosome distribution in standardized cells in two ways that convey complementary information about the results of our experiments: (1) the average pigment distribution map and radial pigment profile for each population of cells (e.g., Figures 1A and 1B) and (2) pigment dispersion distance (PDD) for each cell within a population (e.g., Figure 1C). Pigment maps and radial profiles provide detailed information on the relative distribution of pigment throughout the cytoplasm whereas PDD reports melanosome distribution numerically allowing straightforward statistical comparison of different experimental treatments. Importantly, all experiments (described below) gave similar results when performed using unconstrained melanocytes, indicating that micropatterning does not strongly affect the organization and function of the cytoskeleton. Comparison of nocodazole versus solvent-treated melan-a cells indicated that microtubule depletion had little effect on pigment distribution (mean PDD; DMSO = 19.94 ± 0.6940 μm versus nocodazole = 19.18 ± 0.8312 μm; Figures 1A–1C). Confocal immunofluorescence microscopy (CIFM) analysis using alpha-tubulin-specific antibodies confirmed the efficacy of our nocodazole treatment in depleting microtubules in melanocytes (Figure S1C available online). In contrast, disruption of the actin cytoskeleton using latrunculin A resulted in significant perinuclear clustering of melanosomes, which resembled that seen in immortal myosin-Va-deficient (melan-d1) melanocytes (mean PDD; latrunculin A [25 nM] = 15.80 ± 1.562 μm and melan-d1 11.27 ± 1.682 μm; Figures 1, S1C, and S1D). Interestingly, whereas melanosome clustering was seen over a range of latrunculin A concentrations (5 μM–10 nM), only exposure to low concentrations (<100 nM) that partially depleted F-actin resulted specifically in melanosome clustering without strongly altering cell morphology and attachment (Figures 1, S1C, and S1E).

These observations suggest that a subpopulation of F-actin that is acutely sensitive to latrunculin A is essential for maintaining melanosomes in the peripheral cytoplasm. Given that latrunculin A promotes F-actin depolymerization by forming

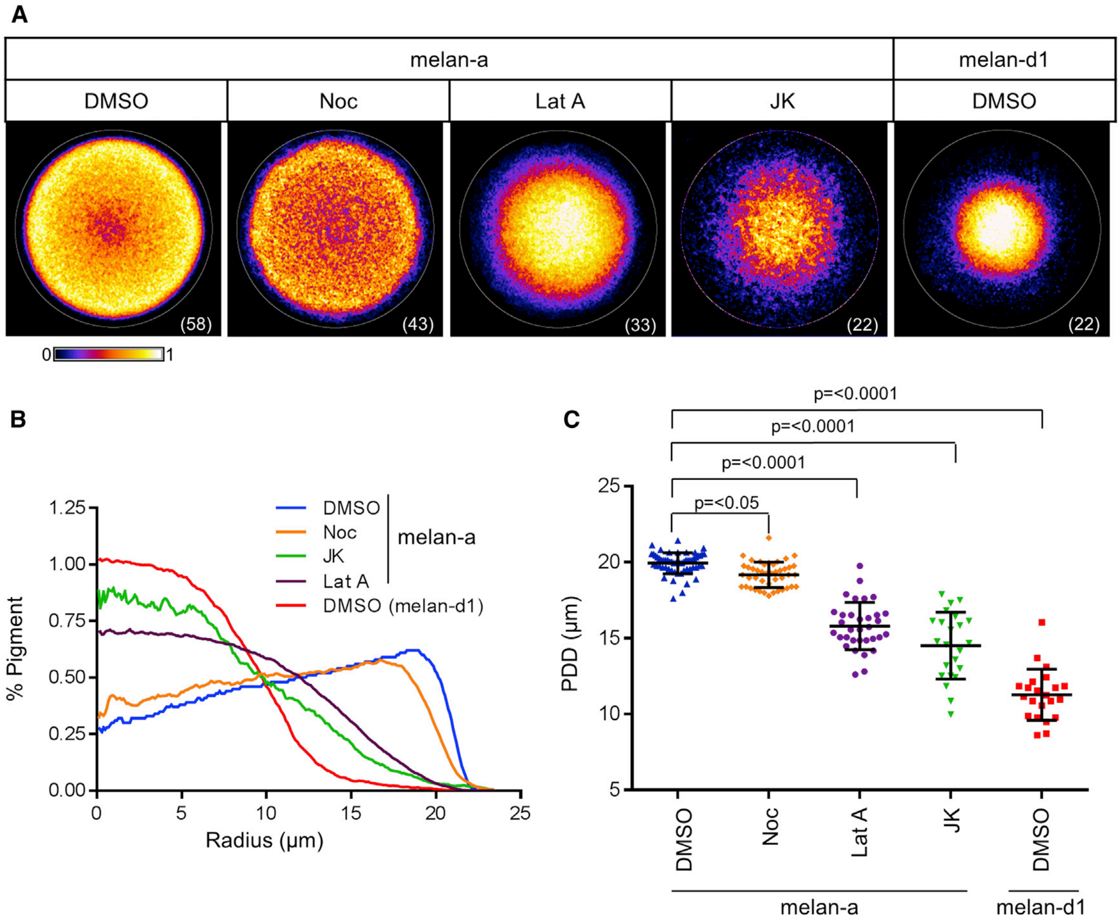

Figure 1. Maintenance of Dispersed Melanosome Distribution in Melanocytes Is Dependent on Actin, but Not Microtubules

Micropattern-grown melan-a and melan-d1 cells were incubated with DMSO 0.1% (solvent control) or cytoskeleton inhibitors for 16 hr (JK, jasplakinolide 8 nM; Lat A, latrunculin A 25 nM; Noc, nocodazole 1 μM).

(A) Probability maps showing melanosome distribution in each population of cells (data are displayed using the fire look up table [LUT]). The white circle and bracketed value in the bottom right-hand corner of each image indicate the border of the micropattern (diameter = 46 μm) and the size of each population analyzed, respectively.

(B and C) Radial profile plots showing the distribution of melanosomes along the average cell radius (B) and scatterplot showing the pigment dispersion distance (PDD) for each cell (C) in each of the populations shown in (A) and (B). Horizontal bars indicate the median and 25th and 75th percentiles of the population in each case. The statistical significance of differences in PDD values between populations is indicated above.

a 1:1 complex with globular (G-)actin, our observations suggested that this population is highly dynamic compared with F-actin involved in maintaining cell morphology and attachment to substrate, which appear to only be affected by higher latrunculin A concentrations (>100 nM) [13]. To further investigate this possibility, we tested the effect of jasplakinolide (8 nM)-induced F-actin stabilization on melanosome distribution [14]. This revealed that, like latrunculin A, jasplakinolide treatment triggered significant melanosome clustering in melan-a cells (mean PDD = 14.51 ± 2.17 μm; Figures 1A–1C). Altogether, these observations suggest an important role for dynamic actin, but not microtubules, in maintaining the dispersed distribution of melanosomes in melanocytes. Mechanistically, this indicates that maintenance of dispersed melanosome distribution requires continuous remodeling of the actin cytoskeleton rather than tethering of organelles to a stable actin cytoskeleton, as envisaged by the cooperative capture model [9].

To investigate the involvement of microtubules in transport, rather than maintenance of dispersion, we tested their role in melanosome redistribution: (1) from dispersed to clustered (centripetal transport) and (2) vice versa (centrifugal transport). For (1), we incubated melan-a cells for 1 hr with nocodazole to deplete microtubules and then for 16 hr with jasplakinolide and nocodazole (JK/Noc) (Figure 2Aii). We then measured the ability of jasplakinolide to cluster melanosomes in these cells and controls in which nocodazole was replaced by solvent (DMSO/JK; Figure 2Ai). This revealed that microtubule depletion significantly reduced jasplakinolide-triggered clustering compared with control cells (mean PDD; Noc/JK = 17.86 ± 1.908 μm versus DMSO/JK = 14.16 ± 2.034 μm; Figures 2Ai and 2Aii). For (2), we preincubated melan-a cells with jasplakinolide for 16 hr to cluster melanosomes and then examined the ability of melanosomes to redisperse after jasplakinolide washout (4 hr) in the presence of solvent (JK/DMSO; Figure 2Aiii) or nocodazole (JK/Noc; Figure 2Aiv). Strikingly, we observed that, not only did melanosomes disperse in microtubule-depleted cells, but that they did so with significantly greater efficiency than in cells with intact microtubules (mean PDD; JK/Noc = 17.63 ± 1.378 μm versus JK/DMSO = 15.20 ± 1.669; Figures

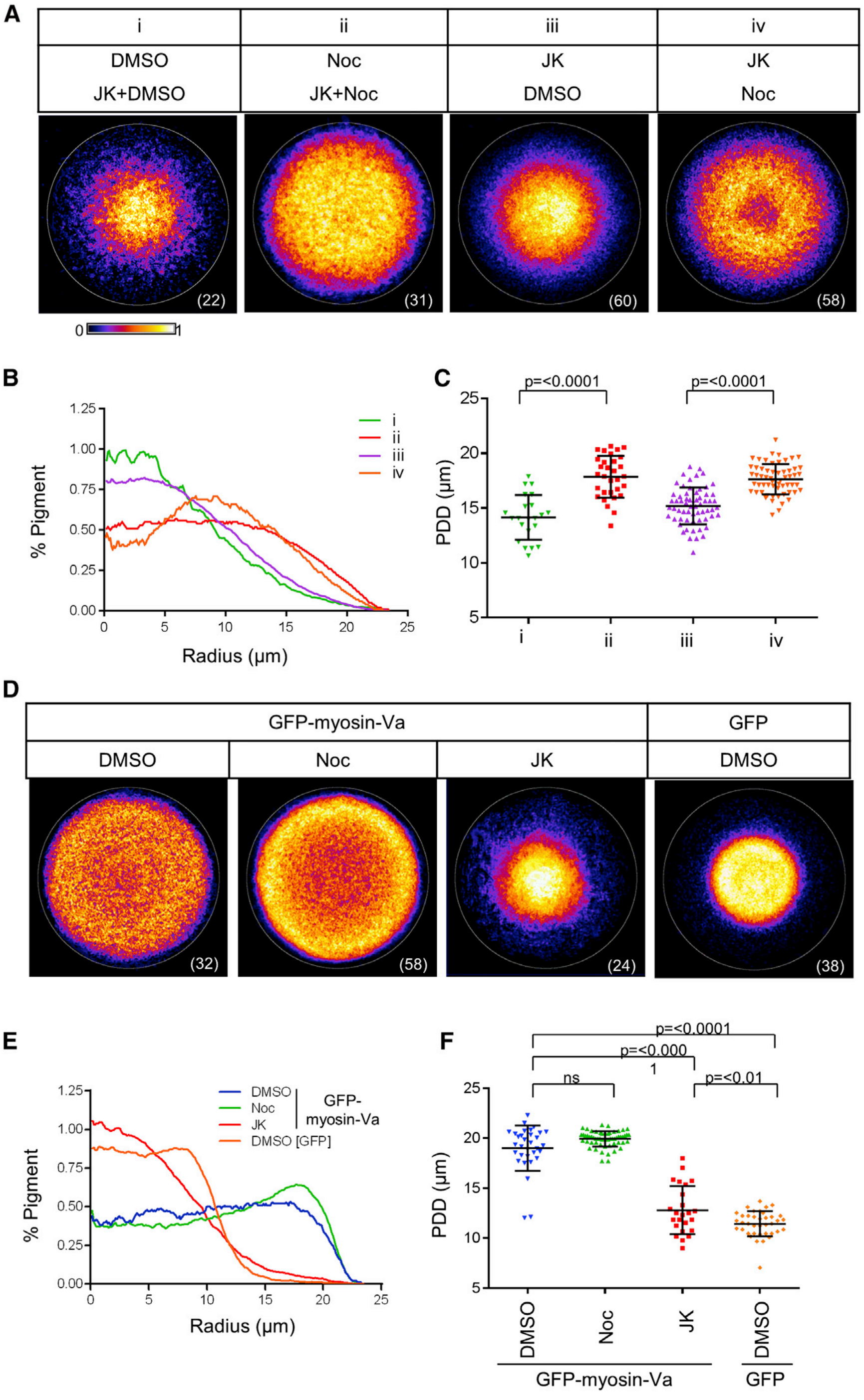

Figure 2. Microtubules Drive Centripetal Melanosome Transport in Opposition to Myosin-Va and Dynamic Actin-Dependent Centrifugal Transport

(A–C) (i and ii) Micropattern-grown melan-a cells were incubated for 1 hr with either DMSO 0.1% (i) or nocodazole 1 μM (ii), then for a further 15 hr with jasplakinolide 8 nM plus either DMSO 0.1% (i) or nocodazole 1 μM (ii), and then fixed. (iii and iv) Melan-a cells were incubated for 15 hr with jasplakinolide 8nM, the inhibitor was then washed out with medium containing either DMSO 0.1% (iii) or nocodazole 1 μM (iv), and fixed 4 hr later.

(D–F) Micropattern-grown melan-d1 cells were incubated with DMSO 0.1%, nocodazole 1 μM, or jasplakinolide 8 nM for 1 hr followed by adenovirus infection and expression of GFP-myosin-Va or GFP for 16 hr in the continued presence of inhibitor or solvent.

(A and D) Probability maps showing melanosome distribution in each population of cells. The white circle and bracketed value in the bottom right-hand corner of each image indicate the border of the micropattern (diameter = 46 μm) and the size of each population analyzed, respectively.

(B and E) Radial profile plots showing the distribution of pigment along the average centroid-to-perimeter radius.

(C and F) Scatterplots showing the PDD for each cell in each of the populations. Horizontal bars are as indicated for Figure 1. The significance of differences in PDD values between populations is indicated above.

2Aiii–2Aiv). These results suggest that microtubules are essential for centripetal, but not centrifugal, melanosome transport.

The finding that dispersion is enhanced in the absence of microtubules (Figures 2Aiii–2Aiv) raises the interesting possibility that the microtubule and actin systems play primarily opposing, rather than cooperative, roles in melanosomes transport. To investigate this using a different and more-targeted approach, we examined the effect of microtubule depletion on the ability of GFP-myosin-Va to complement melanosome clustering in myosin-Va-deficient melan-d1 cells, i.e., drive centrifugal melanosome transport. To do this, we incubated micropattern-grown melan-d1 cells with nocodazole and then added GFP-myosin-Va adenovirus. GFP-myosin-Va was then expressed in the cells in the continued presence of inhibitor for 16 hr. CIFM revealed that GFP-myosin-Va codistributed with melanosomes compared with GFP alone, regardless of microtubule depletion (Figure S2). Comparison of intracellular pigment distribution confirmed that GFP-myosin-Va significantly dispersed the clustered melanosomes compared with GFP alone (mean PDD; GFP-myosin-Va = 18.99 ± 2.262 μm versus GFP = 11.43 ± 1.262 μm; Figures 2D–2F). Moreover, dispersion was greater in microtubule-depleted cells compared with controls (mean PDD = 19.93 ± 0.7691 μm; Figures 2D–2F). In contrast, disruption of F-actin dynamics using jasplakinolide significantly reduced the ability of GFP-myosin-Va to disperse clustered melanosomes in melan-d1 cells (Figures 2D–2F; mean PDD = 12.80 ± 2.409 μm). Similar results were observed on reexpression of GFP-Mlph in Mlph-deficient melan-ln cells. These results further support the idea that myosin-Va and dynamic F-actin drive centrifugal melanosome transport whereas microtubules drive centripetal transport.

The data presented above raised the interesting possibility that myosin-Va functions as an organelle transporter in melanocytes and not a tether as previously suggested [9]. To differentiate between these possibilities, we introduced mutations into GFP-myosin-Va that selectively disrupt isoform-specific

adaptations that allow it to move processively toward the +/barbed ends of F-actin in vitro. We then used the melan-d1 complementation assay to test the functionality of these mutants. The transporter model predicts that characteristics allowing myosin-Va to walk along actin filaments in vitro should be essential for intracellular transport, i.e., ATPase activity, high duty ratio (fraction of the ATPase cycle that myosin-Va remains attached to the actin track), long (36 nm) step size, and +/barbed end directionality, whereas the tether model predicts that myosin-Va should function if it remains competent for actin and cargo binding. Thus, we generated myosin-Va mutants that alter the interaction of myosin-Va with F-actin tracks; the switch II mutant G440A, which increases the affinity and stability of myosin-Va interaction with F-actin by two orders of magnitude [15]; and the switch I mutant S217A, which reduces the duty ratio of myosin-Va from 0.85 to 0.25 [16]. CIFM analysis revealed that the S217A mutant codistributed with melanosomes; however, this was less obvious for the G440A mutant, which appeared more uniformly distributed throughout the cytoplasm, possibly due to its increased affinity for actin (Figures 3B, S3Ai, and S3Aiii). Analysis of melanosome distribution in cells expressing myosin-Va G440A revealed that, rather than restoring dispersion, this mutant caused increased clustering compared with GFP alone in melan-d1 cells (mean PDD; G440A = 10.63 ± 2.193 μm versus GFP = 13.17 ± 1.182 μm; Figure 3). This is likely to be dependent upon attachment to melanosomes and not remodeling of actin as G440A expression did not affect melanosome distribution in melanocytes lacking the melanosomal myosin-Va "receptor" protein Mlph (melan-ln) (Figure S3Bi) [17–20]. In contrast, expression of myosin-Va S217A resulted in melanosome dispersion, albeit to a lesser extent than wild-type, indicating that this mutant retains partial function (mean PDD; S217A = 16.93 ± 2.375 μm versus wild-type [WT] = 18.96 ± 1.354 μm). These data suggest that dynamic interaction with actin is important for myosin-Va function in melanocytes and thus appear consistent with a transporter, and not a tether, mechanism. The surprisingly robust function of the S217A mutant may indicate that multiple myosin-Va molecules cooperate to ensure that melanosomes remain attached to actin tracks during transport.

To further test the transporter role for myosin-Va, we investigated the activity of lever-arm truncation mutants that have reduced step size and velocity in the melan-d1 complementation assay [21, 22]. CIFM analysis revealed that, whereas all proteins codistributed with melanosomes, the 4IQ mutant was also enriched in filopodia-like processes at the cell surface (Figures 3B and S4A). Similar observations of the localization of constitutively active myosin-V were recently reported, suggesting that regulation of this mutant may be compromised [23]. More significantly, analysis of melanosome distribution revealed that the extent of function of the mutants correlated positively with the reduction in lever arm length and expected step size (mean PDD; 4IQ = 15.78 ± 2.694 μm, 2IQ = 14.30 ± 2.057 μm; 0IQ = 12.56 ± 1.830 μm). This pattern of results further supports a transporter function for myosin-Va in melanocytes.

Taking this together with the fact that, in many cell types, the +/barbed ends of actin filaments are oriented toward the plasma membrane, we hypothesized a model in which myosin-Va disperses melanosomes by moving them toward the peripherally located +/barbed ends of actin filaments. To test this directly, we generated chimeric myosin-Va molecules in which the motor domain was replaced with that of the −/pointed-end direct motor myosin-VI (MVI+I/MVa) and then tested their function using the melan-d1 complementation assay [24, 25]. As a control for directionality, we also generated a myosin-VI/Va chimera (MVI−I/MVa) lacking the unique insert (I) that allows movement toward the −/pointed end of actin filaments [26]. After several attempts, we were unable to generate adenoviruses expressing full-length MVI+I/MVa fusions. Therefore, we generated adenoviruses that express "minimyosin" proteins, which comprise the S1 fragment of myosin-Va (i.e., motor and lever arm; see Figure 3A) fused to the Rab27-binding domain (RBD) of synaptotagmin-like protein 2-a (Sytl2a) to allow association with melanosomes (see Supplemental Experimental Procedures) [27]. These were: mini-Va (GFP fused to myosin-Va S1 fused to Sytl2a), mini-VI+I (GFP fused to myosin-VI motor and reverse gear insert fused to myosin-Va lever arm fused to Sytl2-a RBD), and mini-VI−I (as mini-VI+I but lacking I; Figure 4A). Using CIFM, we found that these minimyosin motors all localized efficiently to melanosomes in melan-d1 melanocytes (Figures 4B and S3Aii). Expression of mini-VI+I resulted in increased melanosome clustering in melan-d1 cells compared with GFP alone (mean PDD; mini-VI+I = 10.69 ± 1.54 μm versus GFP = 11.77 ± 1.054 μm; Figures 4B–4D). This "hyperclustering" or "clumping" of pigment was more pronounced than that seen in G440A-expressing cells (Figure 3), was particularly apparent in phase contrast images, and was reflected by comparison of the pigment profile plots (Figures 3, 4B, and 4C; Spearman correlation coefficient; mini-VI+I versus GFP = 0.876, G440A versus GFP = 0.992). Conversely, expression of mini-VI−I protein resulted in increased melanosome dispersion compared with GFP alone (mean PDD = 13.77 ± 2.001 μm; Figures 4B–4D). Nevertheless, this dispersion was less than that resulting from expression of positive control protein mini-Va (mean PDD = 15.35 ± 2.254 μm; Figures 4B–4D), indicating that myosin-VI functions less efficiently than myosin-Va in melanosome transport. Expression of the minimyosins in melanocytes lacking Rab27a (melan-ash), i.e., where they may not attach to melanosomes, resulted in no significant changes in melanosome distribution compared with GFP alone (Figure S3Bii) [27–30]. This indicates that redistribution of melanosomes in melan-d1 results from movement of melanosomes rather than remodeling of the actin cytoskeleton or changes in cell shape. Meanwhile, expression in melan-a cells revealed that all proteins induced significant perinuclear melanosome clustering compared with GFP alone, indicating that they compete for function with endogenous myosin-Va and Mlph (Figure S3Biii). Comparison of the extent of clustering with the RBD alone confirms the activity of the minimyosins in the directional movement of melanosomes. These results support the function of myosin-Va as a transporter dispersing melanosomes by moving them toward the peripherally oriented +/barbed ends of actin filaments. Moreover, as these minimyosins lack the dimerization domain, this further supports the idea that myosin-Va molecules team up to move melanosomes.

Finally, to more directly investigate the role of myosin-Va in melanosome movement, we exploited the rapamycin-induced protein heterodimerization system [31, 32] to generate an acutely activatable myosin-Va. A similar approach was recently used to investigate the role of myosin-V in peroxisome movement [23, 33]. To do this, we divided myosin-Va into two precursors: (1) the S1 (motor/lever arm) fragment fused to human FK506-binding protein 1A (FKBP) and (2) the melanosome-binding tail fused to

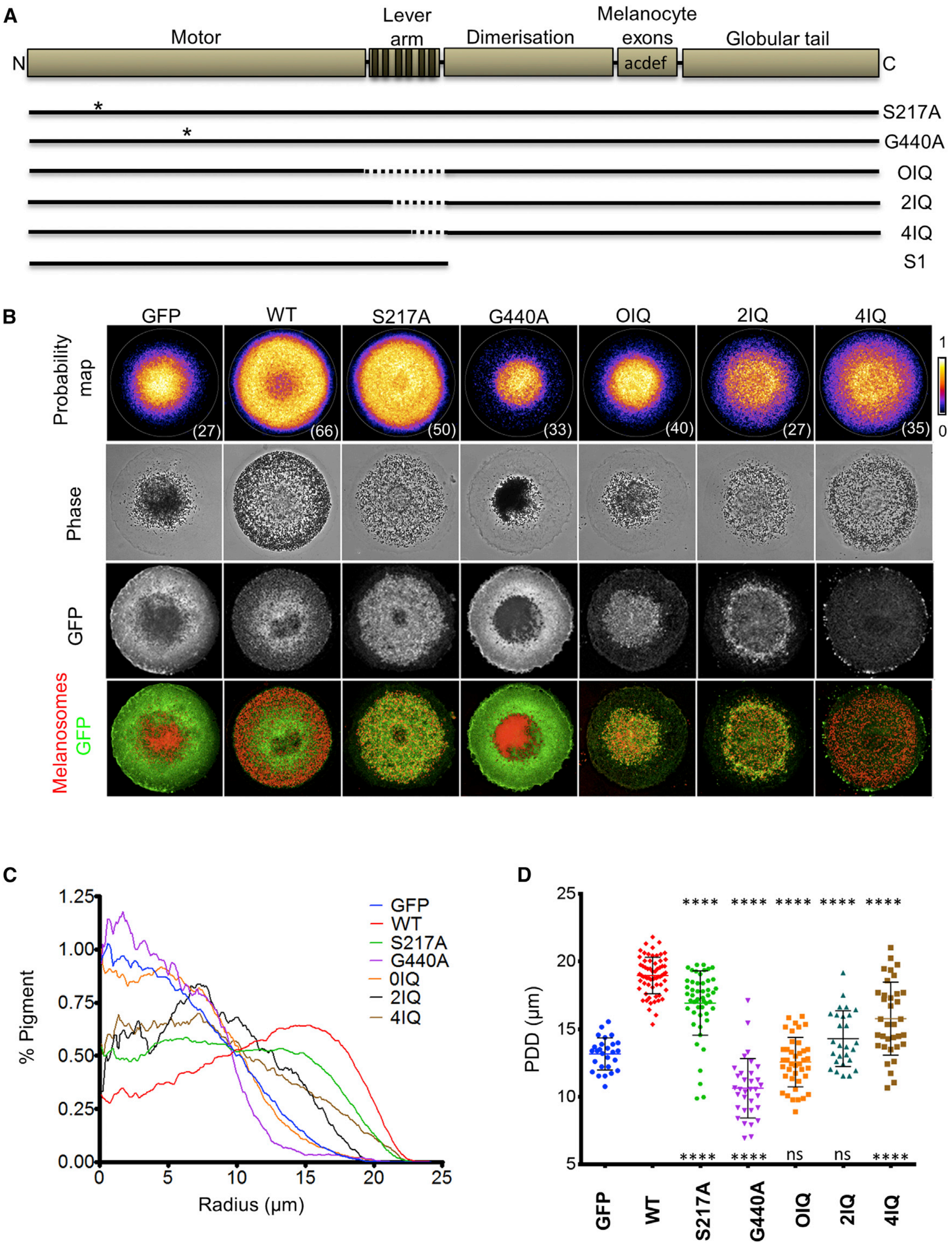

Figure 3. Mutations that Disrupt the Ability of Myosin-Va to Function as a Processive Motor Disrupt Its Function in Melanosome Transport

Melan-d1 cells were infected with adenovirus vectors, allowing expression of GFP-myosin-Va wild-type and mutant variants or GFP alone, plated onto micropattern and then fixed and processed for immunofluorescence.

(A) A schematic representation of the domain organization of myosin-Va (block diagram) shows the position of point mutations (asterisks) and internal deletions (dashed lines; line diagram).

(B) Probability maps (top row, displayed with fire LUT) for each population of cells (n of each indicated in bottom right) and representative images of GFP and melanosome distribution in individual cells from each population. White circles (top row) indicate the shape of the micropattern (diameter = 46 μm).

(C) Radial profile plots showing the distribution of pigment along the average cell radius.

(D) Scatterplot showing the PDD for each cell in each of the populations. Horizontal bars are as indicated for Figure 1. The significance of differences in PDD values for each mutant compared with the GFP and wild-type myosin-Va are displayed below and above each scatter, respectively. ****$p < 0.0001$.

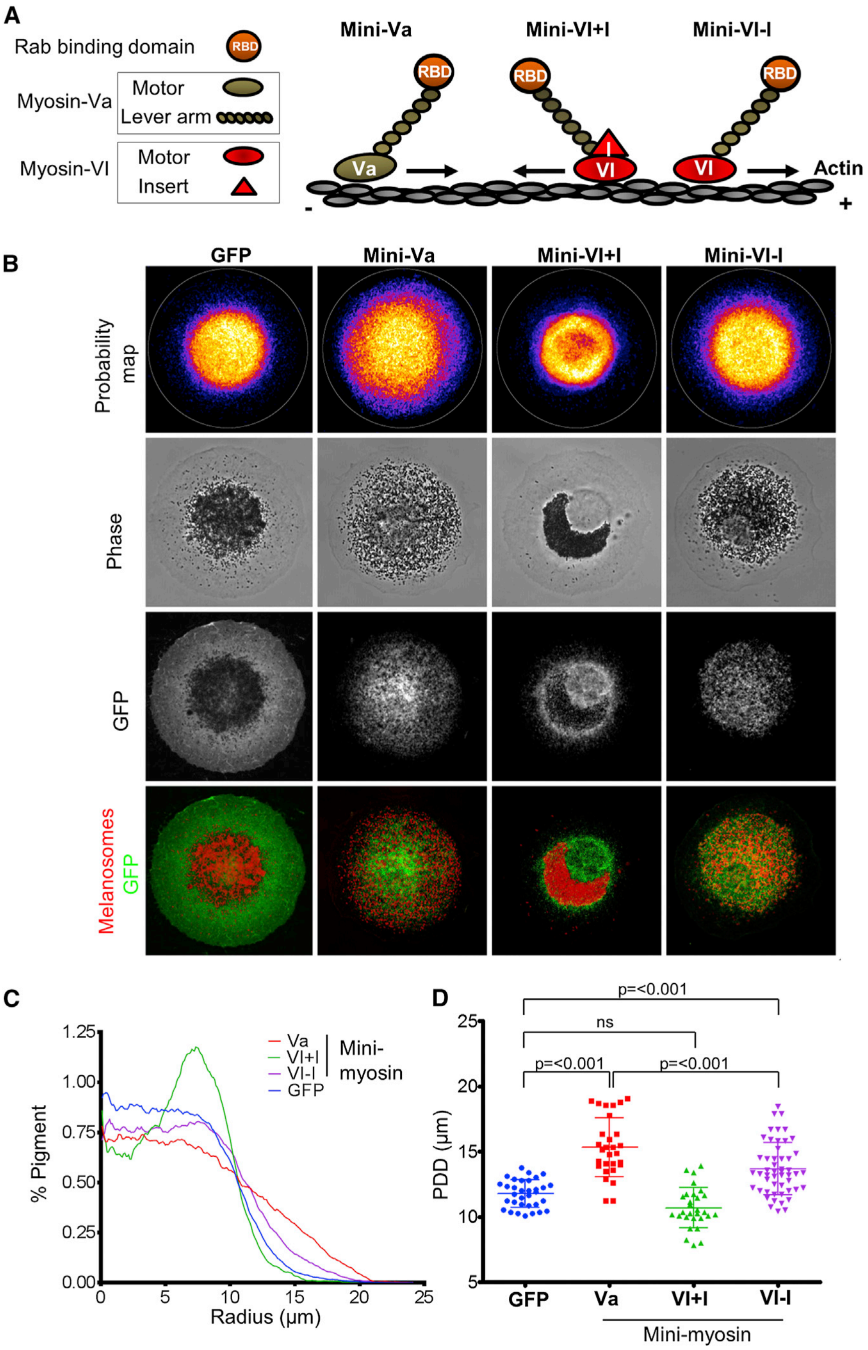

Figure 4. Myosin-Va Disperses Melanosomes in Melanocytes by Moving Them toward the Peripherally Oriented +/Barbed Ends of Actin Filaments

Melan-d1 cells were infected with adenovirus vectors, allowing expression of the indicated minimyosin proteins, plated onto micropatterns, fixed, and stained for immunofluorescence (as described in Experimental Procedures).

(A) A schematic representation of the structure of the minimyosin molecules tested. Arrows indicate directionality of the protein, orange RBD-labeled circles indicate Rab27a-binding domain of murine Sytl2-a (GenBank accession no. NM_001289583.1; amino acids 1–90), red shapes indicate the motor (VI; amino acids 1–771) and unique insert (I; amino acids 772–809) of human myosin-VI (GenBank accession no. NM_004999.3), and the brown shapes indicate the motor (Va; amino acids 1–762) and lever arm (amino acids 763–920) of murine myosin-Va (GenBank accession no. NM_010864).

(B) Probability maps (top row, displayed with fire LUT) for each population of cells (n of each indicated in bottom right) and representative images of GFP and melanosome distribution in individual cells from each population. White circles (top row) indicate the shape of the micropattern (diameter = 46 µm).

(C) Radial profile plots showing the distribution of pigment along the average cell radius.

(D) Scatterplot showing the PDD for each cell in each of the populations. Horizontal bars are as indicated for Figure 1. The significance of differences in PDD values between populations is indicated above.

FKBP-rapamycin-binding domain (hereafter S1 and tail; see Figure S4a and Supplemental Experimental Procedures). In live cells, these interact exclusively upon addition of A/C heterodimerizer to reconstitute active myosin-Va. Confocal microscopy confirmed that the tail localized to clustered melanosomes and the S1 was distributed throughout the cytoplasm in melan-d1 before addition of A/C heterodimerizer. On addition of A/C heterodimerizer, melanosomes moved centrifugally en masse into the peripheral cytoplasm within 60 min and without obvious changes in cells' shapes (Figures S4B and S4C; Movie S1). Microtubule depletion did not perturb this redistribution, confirming that myosin-Va can drive long-distance centrifugal melanosome transport in melanocytes without assistance from microtubule motors (Figures S4B and S4C).

In this study, we investigated how the actin and microtubule networks regulate organelle transport using the melanosomes of melanocytes as a model. Consistent with the previous study of the role of the cytoskeleton in transport in melanocytes, we found that microtubules were essential for melanosome clustering/centripetal transport [9]. In contrast to the prediction of the cooperative capture model, however, we found that microtubules were nonessential for centrifugal transport or maintenance of dispersed melanosomes [9]. Instead, we found that these processes were dependent upon myosin-Va and a subpopulation of dynamic actin. This indicates that myosin-Va and dynamic actin comprise a transport system and not a tether as suggested previously [9]. In accord with this, we showed that the ability of myosin-Va to function in melanosome transport correlated with its ability to act as a motor moving toward the +/barbed ends of actin filaments. Overall, our data provide further support for the emerging view that actin-myosin networks represent a conserved mechanism mediating long-distance organelle transport [11]. Our results are consistent with those of earlier studies of melanosome transport in amphibian melanophores [34, 35]. Based on our results, we propose a new model for transport in which melanosome distribution is

determined by the balance of two forces: microtubule-dependent centripetal and myosin-Va/dynamic-actin-dependent centrifugal transport. More specifically, we suggest that myosin-Va works in opposition to microtubule-dependent centripetal transport as a processive motor transporting melanosomes toward the cell periphery along a network of dynamic actin tracks whose +/barbed ends are overall oriented toward the plasma membrane. Consistent with this, disruption of myosin-Va/dynamic actin results in microtubule-dependent clustering whereas depletion of microtubules allows myosin-Va/dynamic actin to hyperdisperse melanosomes. However, at this stage, precisely how this process of myosin-Va/actin-dependent transport process occurs is unclear and we cannot exclude the possibility that a more-complex process might drive centrifugal melanosome transport. Along these lines, one possibility is that the rigidity of the melanin core necessitates that melanosome transport is coupled to a certain level of remodelling of the thin peripheral cytoplasm. This might be more efficiently achieved by an actin-dependent mechanism, given the relatively isotropic distribution of actin filaments compared with microtubules. Nevertheless, video microscopy shows that some melanosomes move bidirectionally on microtubules, i.e., microtubule-based centrifugal transport can occur [9, 36]. This may be important for the maturation of melanosomes rather than the transport of mature melanosomes that predominate in melan-a cells.

Finally, how does our model fit with the physiological role of the melanocyte in transfer of pigment to keratinocytes? Although the mechanism in situ remains poorly characterized, recent live-cell studies of cocultures indicate that melanocytes deliver pigment to keratinocytes via a process involving dendrite extension, adhesion, thinning, and breakage, followed by phagocytosis of the severed dendrite tip by keratinocytes [37]. Strikingly, time-lapse image sequences from this study appear to show that wild-type melanocytes dynamically extend pigment-filled dendrites (on a minutes to hours timescale) rather than pigment-free dendrites that are then filled with pigment via long-range transport from the cell body. Given the relatively slow rate of actin-myosin-Va-dependent transport in mammalian cells (similar to dendrite extension), these observations appear consistent with our model for myosin-actin as the main mechanism driving centrifugal transport. In this context, we suggest that actin-myosin-Va-dependent transport could ensure dendrite extension, likely driven by actin polymerization in response to keratinocyte-derived paracrine signals, and melanosome accumulation is coordinated, leading to the formation of pigment-filled dendrites and efficient transfer to keratinocytes.

Experimental Procedures

Micropattern Cells

For experiments using cytoskeleton inhibitor treatments with or without adenovirus (Figures 1 and 2), cells (2×10^4) were seeded into wells of 24-well cluster plates containing CYTOOchips Mini DC-L-FN650 (Cytoo 11-003-13-12). After cells had achieved full spreading (4 hr), they were treated with inhibitors (and adenovirus) and then fixed and immunostained as described above. For melan-d1 complementation assays measuring the function of myosin mutants (Figures 3 and 4), cells were seeded in a 24-well plate and allowed to reattach prior to infection with adenovirus. After 96 hr, cells were trypsinized and (2×10^4) transferred onto a Cytoo minichip. After cells had achieved full spreading (4 hr), they were fixed and immunostained as described above. Cells were imaged on a Zeiss LSM 710 confocal microscope as above. Typically, between 25 and 50 cells per experiment were manually located and then automatically acquired with autofocus on the micropatterns (excitation 633 nm; emission 650 nm). All image processing and analysis was performed using NIH Image J. Images were aligned in reference to the micropattern using a modified macro available from Cytoo (CellRefDemo). Pigment distribution was measured by applying a threshold to the phase-contrast images for low pixel intensity so as to only include dark-pigmented melanosomes; other high refractive index organelles such as autophagosomes, lysosomes, and lipid droplets were excluded by this threshold (Figure S1A). The pigment image was then converted to binary and inverted. Averaging the binary images of individual cells in a stack was used to make average pigment probability maps. Melanosome distribution was calculated by determining the radial profile of the binary images (Radial Profile Extended; http://rsbweb.nih.gov/ij/plugins/radial-profile-ext.html). The PDD was defined as the distance from the center of the micropattern at which 95% of the area under the curve was accounted for. Statistical analysis of data was carried out with GraphPad Prism 6 software using the one-way ANOVA test and Bonferroni's multiple comparisons posttest facility within the software and assuming nonparametric distribution of data.

Supplemental Information

Supplemental Information includes Supplemental Experimental Procedures, four figures, and one movie and can be found with this article online at http://dx.doi.org/10.1016/j.cub.2014.06.019.

Author Contributions

R.D.E., C.R., D.A.B., D.J.T., and A.N.H. conducted the experiments. J.S.R., M.C., L.M., S.P., and E.V.S. contributed novel reagents. A.N.H., R.D.E., and C.R. designed the studies and wrote the manuscript.

Acknowledgments

We would like to thank Miguel Seabra (Imperial College London) for supplying dilute20j mice; Folma Buss (University of Cambridge) for the human myosin VI cDNA; Sally Wheatley for LC3 antibodies and chloroquine; Andrew Bennett for Nile Red (both University of Nottingham); and David Onion (flow cytometry), Tim Self (confocal microscopy), and Sue Cooper and Carol Sculthorpe (general) (all University of Nottingham) for technical assistance. A.N.H., R.D.E., and D.A.B. were supported by a Medical Research Council New Investigator Award (grant reference G1100063); C.R. was supported by the Biotechnology and Biological Sciences Research Council and a University of Nottingham funded PhD studentship; L.M. was supported by CIBERER-ISCIII, Biomedicine project "VISIONANIMAL," Comunidad de Madrid, S2010/BMD-2439; and S.P. was supported by a SWan (the South-West London Academic Network) funded PhD studentship.

Received: March 22, 2014
Revised: May 6, 2014
Accepted: June 10, 2014
Published: July 24, 2014

References

1. Goode, B.L., Drubin, D.G., and Barnes, G. (2000). Functional cooperation between the microtubule and actin cytoskeletons. Curr. Opin. Cell Biol. *12*, 63–71.
2. Langford, G.M. (1995). Actin- and microtubule-dependent organelle motors: interrelationships between the two motility systems. Curr. Opin. Cell Biol. *7*, 82–88.
3. Ross, J.L., Ali, M.Y., and Warshaw, D.M. (2008). Cargo transport: molecular motors navigate a complex cytoskeleton. Curr. Opin. Cell Biol. *20*, 41–47.
4. Alberts, B., Johnson, A., Lewis, J., Raff, M., Roberts, K., and Walter, P. (2008). Molecular Biology of the Cell, Fifth Edition (New York: Garland Science).
5. Hammer, J.A., 3rd, and Sellers, J.R. (2012). Walking to work: roles for class V myosins as cargo transporters. Nat. Rev. Mol. Cell Biol. *13*, 13–26.
6. Hirokawa, N., Noda, Y., Tanaka, Y., and Niwa, S. (2009). Kinesin superfamily motor proteins and intracellular transport. Nat. Rev. Mol. Cell Biol. *10*, 682–696.
7. Kardon, J.R., and Vale, R.D. (2009). Regulators of the cytoplasmic dynein motor. Nat. Rev. Mol. Cell Biol. *10*, 854–865.

8. Woolner, S., and Bement, W.M. (2009). Unconventional myosins acting unconventionally. Trends Cell Biol. *19*, 245–252.
9. Wu, X., Bowers, B., Rao, K., Wei, Q., and Hammer, J.A., 3rd. (1998). Visualization of melanosome dynamics within wild-type and dilute melanocytes suggests a paradigm for myosin V function In vivo. J. Cell Biol. *143*, 1899–1918.
10. Pruyne, D., Legesse-Miller, A., Gao, L., Dong, Y., and Bretscher, A. (2004). Mechanisms of polarized growth and organelle segregation in yeast. Annu. Rev. Cell Dev. Biol. *20*, 559–591.
11. Schuh, M. (2011). An actin-dependent mechanism for long-range vesicle transport. Nat. Cell Biol. *13*, 1431–1436.
12. Hume, A.N., Tarafder, A.K., Ramalho, J.S., Sviderskaya, E.V., and Seabra, M.C. (2006). A coiled-coil domain of melanophilin is essential for Myosin Va recruitment and melanosome transport in melanocytes. Mol. Biol. Cell *17*, 4720–4735.
13. Coué, M., Brenner, S.L., Spector, I., and Korn, E.D. (1987). Inhibition of actin polymerization by latrunculin A. FEBS Lett. *213*, 316–318.
14. Bubb, M.R., Senderowicz, A.M., Sausville, E.A., Duncan, K.L., and Korn, E.D. (1994). Jasplakinolide, a cytotoxic natural product, induces actin polymerization and competitively inhibits the binding of phalloidin to F-actin. J. Biol. Chem. *269*, 14869–14871.
15. Yengo, C.M., De la Cruz, E.M., Safer, D., Ostap, E.M., and Sweeney, H.L. (2002). Kinetic characterization of the weak binding states of myosin V. Biochemistry *41*, 8508–8517.
16. Forgacs, E., Sakamoto, T., Cartwright, S., Belknap, B., Kovács, M., Tóth, J., Webb, M.R., Sellers, J.R., and White, H.D. (2009). Switch 1 mutation S217A converts myosin V into a low duty ratio motor. J. Biol. Chem. *284*, 2138–2149.
17. Fukuda, M., Kuroda, T.S., and Mikoshiba, K. (2002). Slac2-a/melanophilin, the missing link between Rab27 and myosin Va: implications of a tripartite protein complex for melanosome transport. J. Biol. Chem. *277*, 12432–12436.
18. Hume, A.N., Collinson, L.M., Hopkins, C.R., Strom, M., Barral, D.C., Bossi, G., Griffiths, G.M., and Seabra, M.C. (2002). The leaden gene product is required with Rab27a to recruit myosin Va to melanosomes in melanocytes. Traffic *3*, 193–202.
19. Provance, D.W., James, T.L., and Mercer, J.A. (2002). Melanophilin, the product of the leaden locus, is required for targeting of myosin-Va to melanosomes. Traffic *3*, 124–132.
20. Wu, X.S., Rao, K., Zhang, H., Wang, F., Sellers, J.R., Matesic, L.E., Copeland, N.G., Jenkins, N.A., and Hammer, J.A., 3rd. (2002). Identification of an organelle receptor for myosin-Va. Nat. Cell Biol. *4*, 271–278.
21. Sakamoto, T., Wang, F., Schmitz, S., Xu, Y., Xu, Q., Molloy, J.E., Veigel, C., and Sellers, J.R. (2003). Neck length and processivity of myosin V. J. Biol. Chem. *278*, 29201–29207.
22. Schott, D.H., Collins, R.N., and Bretscher, A. (2002). Secretory vesicle transport velocity in living cells depends on the myosin-V lever arm length. J. Cell Biol. *156*, 35–39.
23. Kapitein, L.C., van Bergeijk, P., Lipka, J., Keijzer, N., Wulf, P.S., Katrukha, E.A., Akhmanova, A., and Hoogenraad, C.C. (2013). Myosin-V opposes microtubule-based cargo transport and drives directional motility on cortical actin. Curr. Biol. *23*, 828–834.
24. Buss, F., Spudich, G., and Kendrick-Jones, J. (2004). Myosin VI: cellular functions and motor properties. Annu. Rev. Cell Dev. Biol. *20*, 649–676.
25. Sweeney, H.L., and Houdusse, A. (2010). Myosin VI rewrites the rules for myosin motors. Cell *141*, 573–582.
26. Park, H., Li, A., Chen, L.Q., Houdusse, A., Selvin, P.R., and Sweeney, H.L. (2007). The unique insert at the end of the myosin VI motor is the sole determinant of directionality. Proc. Natl. Acad. Sci. USA *104*, 778–783.
27. Kuroda, T.S., Fukuda, M., Ariga, H., and Mikoshiba, K. (2002). The Slp homology domain of synaptotagmin-like proteins 1-4 and Slac2 functions as a novel Rab27A binding domain. J. Biol. Chem. *277*, 9212–9218.
28. Bahadoran, P., Aberdam, E., Mantoux, F., Buscà, R., Bille, K., Yalman, N., de Saint-Basile, G., Casaroli-Marano, R., Ortonne, J.P., and Ballotti, R. (2001). Rab27a: A key to melanosome transport in human melanocytes. J. Cell Biol. *152*, 843–850.
29. Hume, A.N., Collinson, L.M., Rapak, A., Gomes, A.Q., Hopkins, C.R., and Seabra, M.C. (2001). Rab27a regulates the peripheral distribution of melanosomes in melanocytes. J. Cell Biol. *152*, 795–808.
30. Wu, X., Rao, K., Bowers, M.B., Copeland, N.G., Jenkins, N.A., and Hammer, J.A., 3rd. (2001). Rab27a enables myosin Va-dependent melanosome capture by recruiting the myosin to the organelle. J. Cell Sci. *114*, 1091–1100.
31. Rivera, V.M., Clackson, T., Natesan, S., Pollock, R., Amara, J.F., Keenan, T., Magari, S.R., Phillips, T., Courage, N.L., Cerasoli, F., Jr., et al. (1996). A humanized system for pharmacologic control of gene expression. Nat. Med. *2*, 1028–1032.
32. Castellano, F., Montcourrier, P., Guillemot, J.C., Gouin, E., Machesky, L., Cossart, P., and Chavrier, P. (1999). Inducible recruitment of Cdc42 or WASP to a cell-surface receptor triggers actin polymerization and filopodium formation. Curr. Biol. *9*, 351–360.
33. Efremov, A.K., Radhakrishnan, A., Tsao, D.S., Bookwalter, C.S., Trybus, K.M., and Diehl, M.R. (2014). Delineating cooperative responses of processive motors in living cells. Proc. Natl. Acad. Sci. USA *111*, E334–E343.
34. Rogers, S.L., and Gelfand, V.I. (1998). Myosin cooperates with microtubule motors during organelle transport in melanophores. Curr. Biol. *8*, 161–164.
35. Gross, S.P., Tuma, M.C., Deacon, S.W., Serpinskaya, A.S., Reilein, A.R., and Gelfand, V.I. (2002). Interactions and regulation of molecular motors in Xenopus melanophores. J. Cell Biol. *156*, 855–865.
36. Hume, A.N., Wilson, M.S., Ushakov, D.S., Ferenczi, M.A., and Seabra, M.C. (2011). Semi-automated analysis of organelle movement and membrane content: understanding rab-motor complex transport function. Traffic *12*, 1686–1701.
37. Wu, X.S., Masedunskas, A., Weigert, R., Copeland, N.G., Jenkins, N.A., and Hammer, J.A. (2012). Melanoregulin regulates a shedding mechanism that drives melanosome transfer from melanocytes to keratinocytes. Proc. Natl. Acad. Sci. USA *109*, E2101–E2109.

Current Biology, Volume *24*
Supplemental Information

Myosin-Va and Dynamic Actin Oppose Microtubules to Drive Long-Range Organelle Transport

Richard D. Evans, Christopher Robinson, Deborah A. Briggs, David J. Tooth,
Jose S. Ramalho, Marta Cantero, Lluis Montoliu, Shyamal Patel, Elena V. Sviderskaya,
and Alistair N. Hume

Figure S1a-b

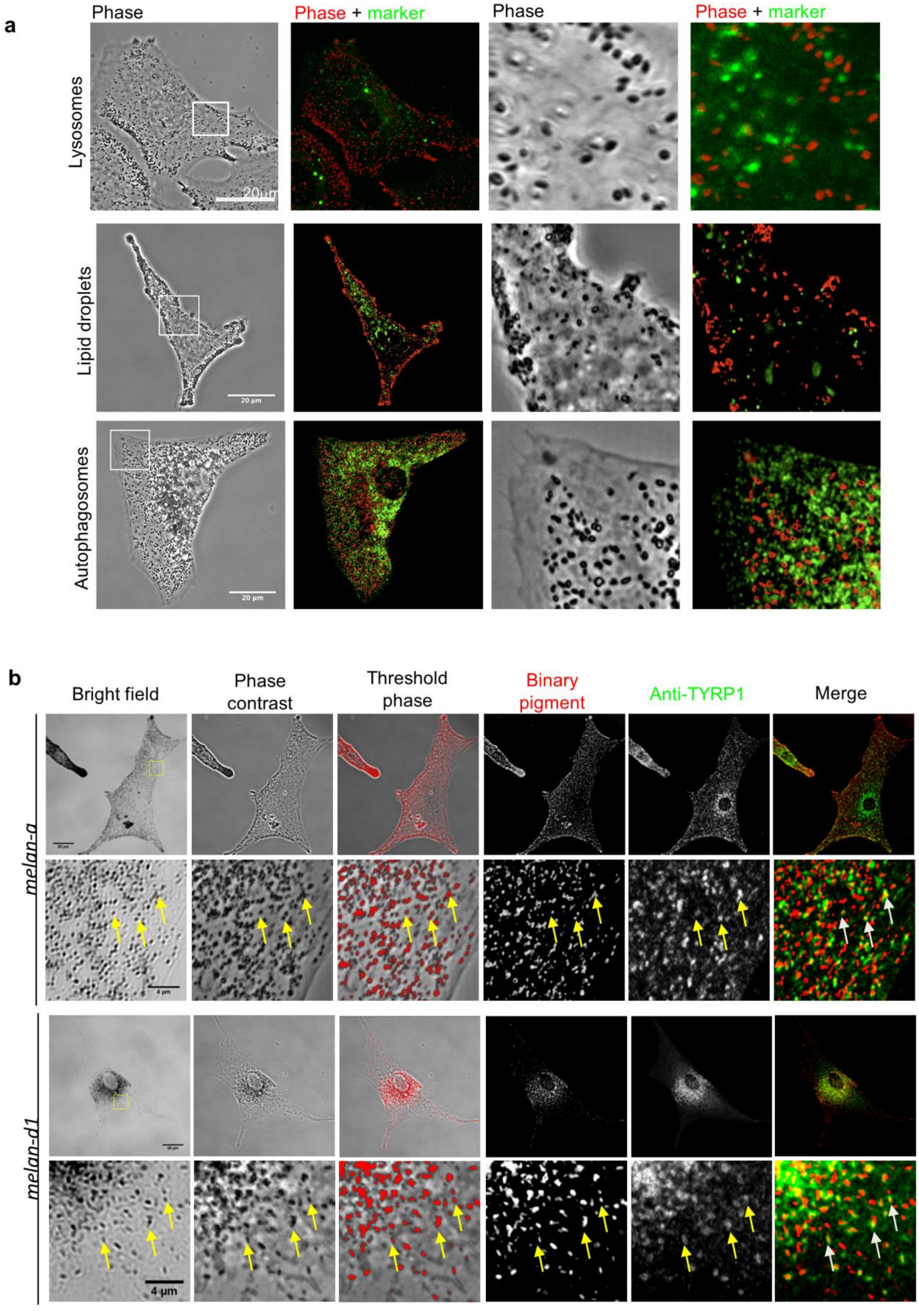

a
Phase
Phase + marker
Phase
Phase + marker
Lysosomes
Lipid droplets
Autophagosomes
20μm
20 μm
20 μm
b
Bright field
Phase contrast
Threshold phase
Binary pigment
Anti-TYRP1
Merge
melan-a
melan-d1
4 μm

Figure S1c-e

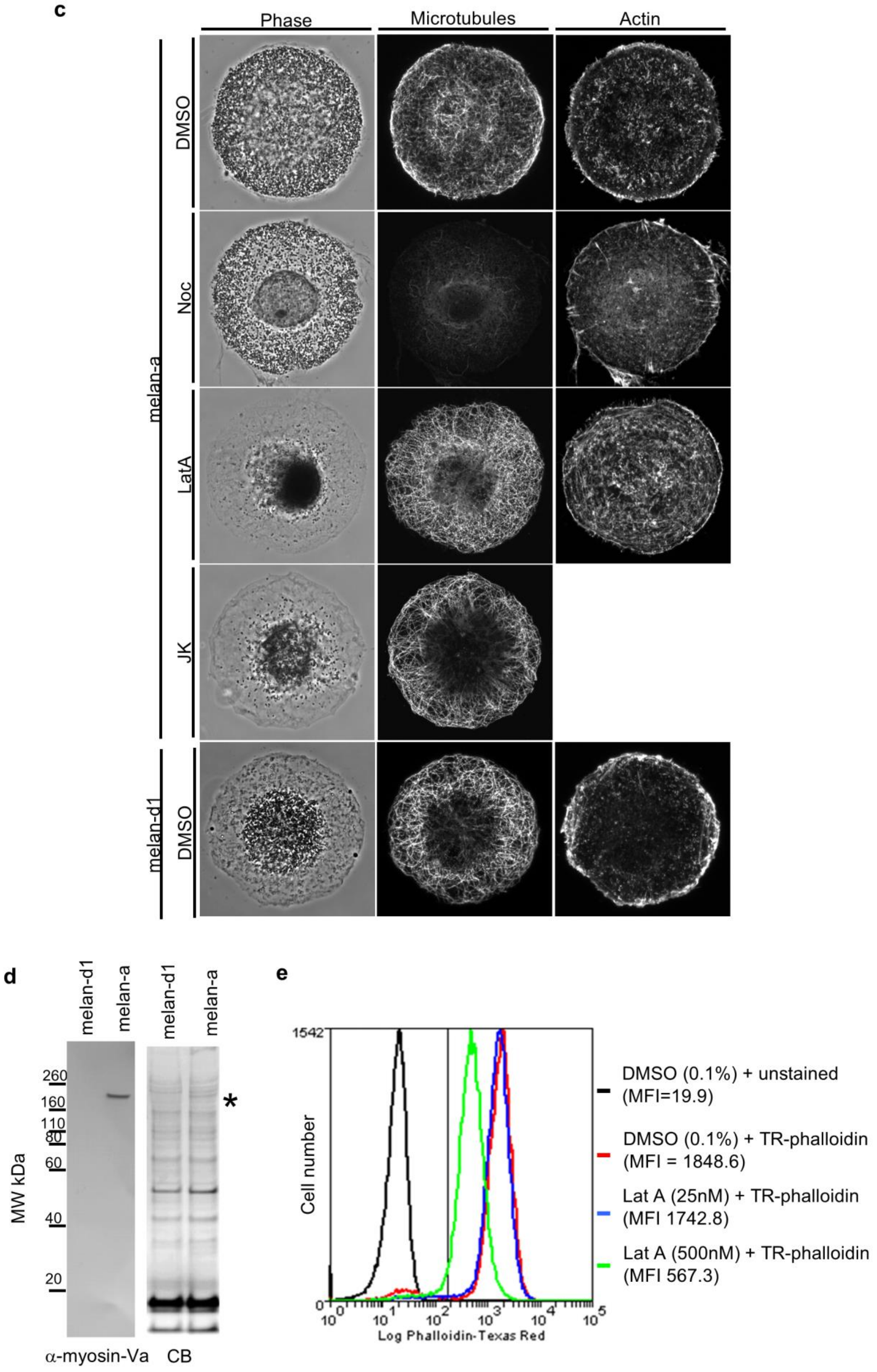

c
Phase
Microtubules
Actin
melan-a
DMSO
Noc
LatA
JK
melan-d1
DMSO
d
melan-d1
melan-a
melan-d1
melan-a
260
160
110
80
60
40
20
MW kDa
*
α-myosin-Va
CB
e
1542
Cell number
Log Phalloidin-Texas Red
DMSO (0.1%) + unstained (MFI=19.9)
DMSO (0.1%) + TR-phalloidin (MFI = 1848.6)
Lat A (25nM) + TR-phalloidin (MFI 1742.8)
Lat A (500nM) + TR-phalloidin (MFI 567.3)

Figure S2

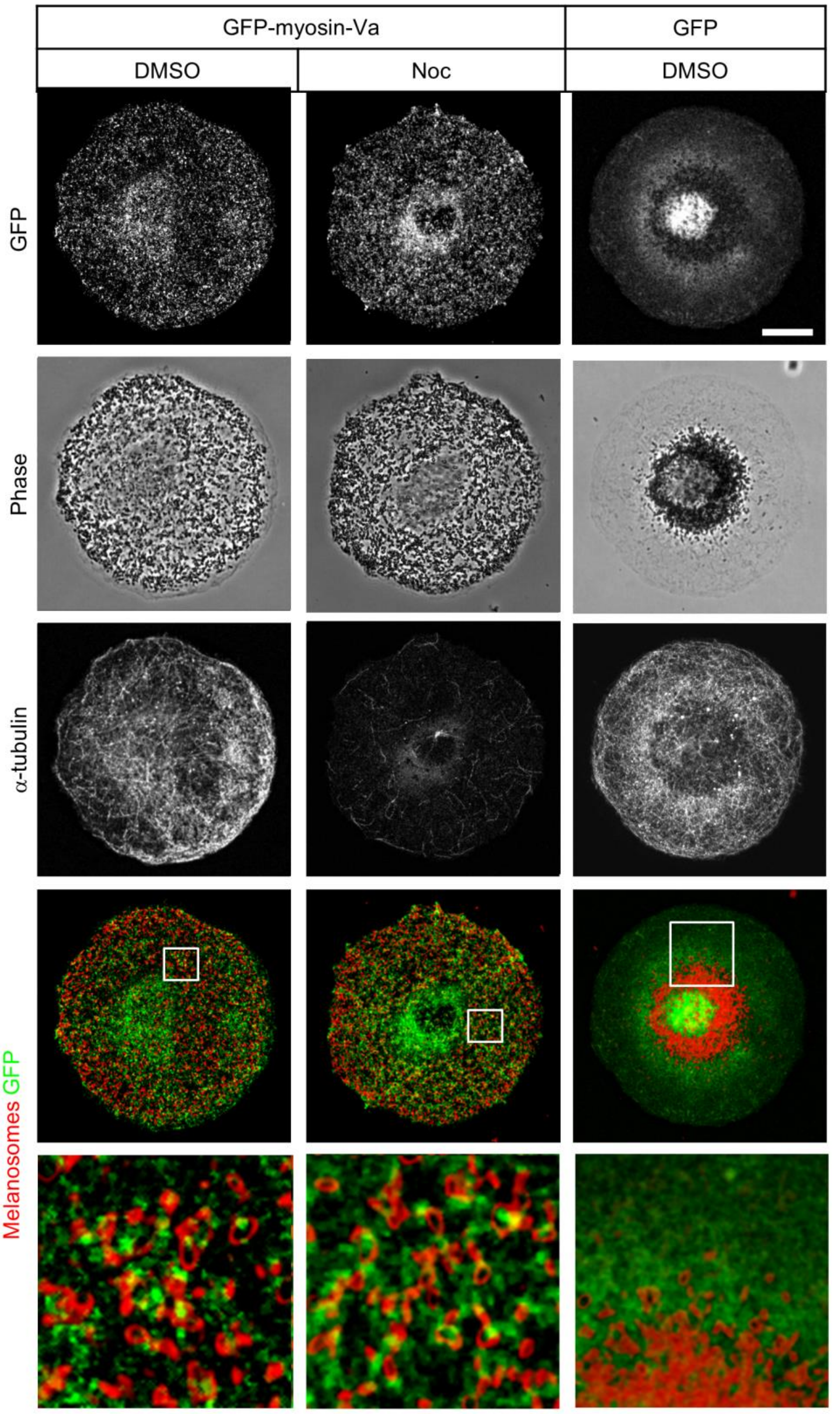
GFP-myosin-Va
GFP
DMSO
Noc
DMSO
GFP
Phase
α-tubulin
Melanosomes GFP

Figure S3a

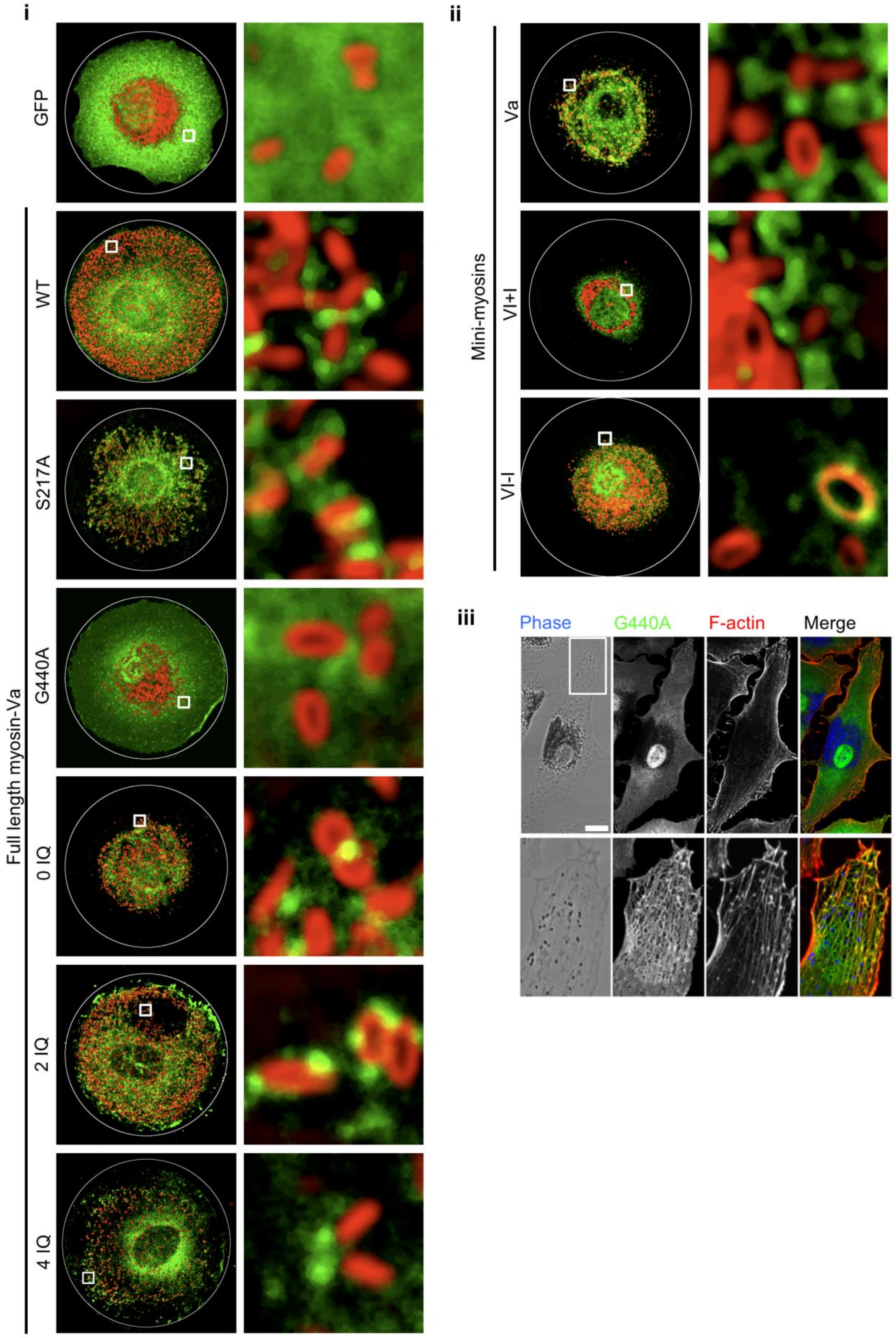
i
GFP
WT
S217A
G440A
0 IQ
2 IQ
4 IQ
Full length myosin-Va
ii
Va
VI+I
VI-I
Mini-myosins
iii
Phase
G440A
F-actin
Merge

Figure S3b

i) melan-ln

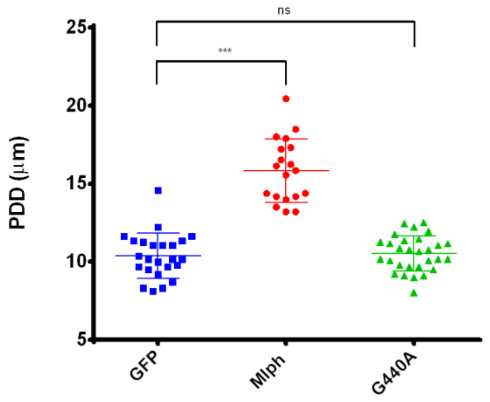

ii) melan-ash

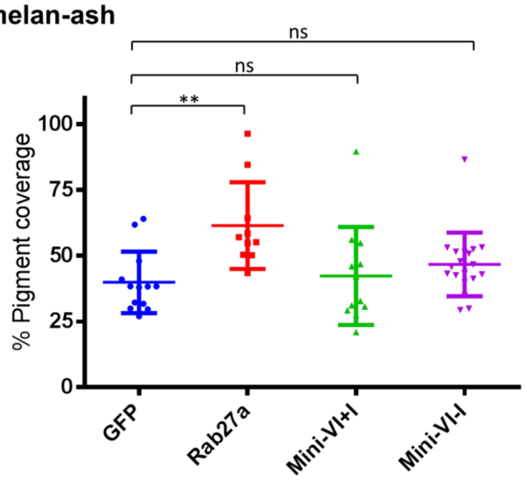

iii) melan-a

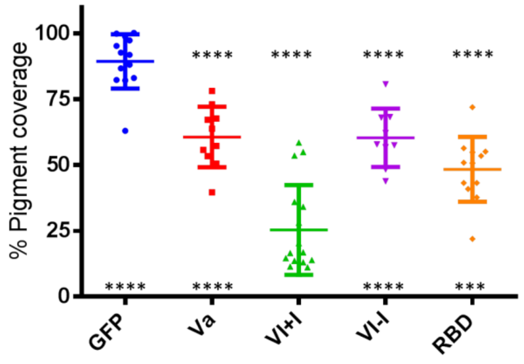

Figure S4

a

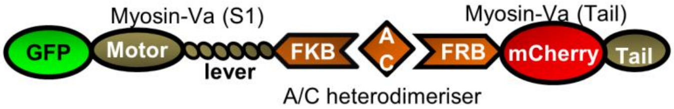

b

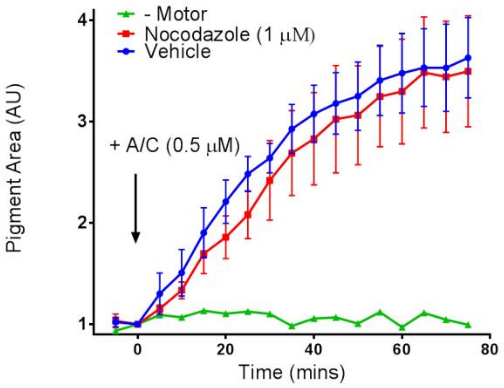

c

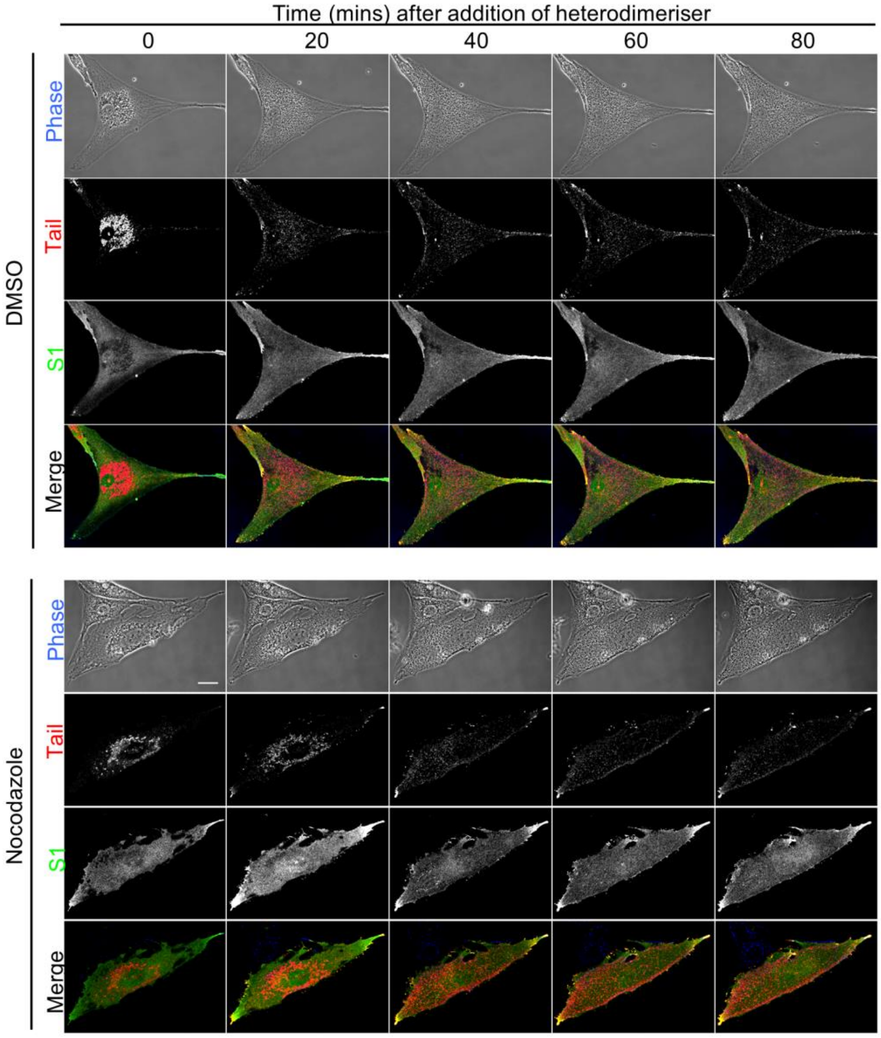

Supplemental figure legends.

Figure S1 (companion to main figure 1). a) The localisation of highly refractive organelles; lysosomes, lipid droplets and autophagosomes relative to pigmented melanosomes was revealed by lysotracker red, nile red (staining on live cells) and LC3 (staining on chloroquine treated (120mM for 4 hours) fixed cells) staining of melan-a cells. Images are single confocal z-sections at the bottom of the cell where most melanosomes are located. This confirmed that the majority of organelles analysed in this study were melanosomes. b) Tyrosinase-related protein-1 localisation in melan-a and melan-d1 melanocytes, upper and lower rows, respectively. Melanocytes were fixed and stained with rabbit anti-Tyrp-1 (provided by Vince Hearing from the US National Cancer Institute) and then images of melanosomes (bright-field (leftmost column) and phase contrast (second column)) and Tyrp-1 (fifth column) distribution were acquired using a confocal microscope. In the third column we show in red the structures in the phase contrast images (second column) which were included in the binary pigment maps (fourth column). In the rightmost (sixth) column we show the extent of co-localisation between structures in the binary pigment map and Tyrp-1 (highlighted by arrows). Boxes in low magnification images (upper rows) indicate the area presented in the high magnification images (lower rows). Bars = 20μm and 4μm for high and low magnification images, respectively. (c) The distribution of cytoskeleton networks in micro-pattern-grown melanocytes and the effects of cytoskeleton inhibitors. Micro-pattern-grown melan-a and melan-d1 cells were incubated with the indicated cytoskeleton inhibitors or solvent control (0.1% DMSO), fixed and stained with phalloidin and alpha-tubulin-specific antibodies (as described in Experimental procedures). Left-hand, middle and right-hand panels are images showing the distribution of melanosomes (phase contrast), microtubules and actin filaments, respectively. The F-actin distribution in jasplakinolide treated cells is not shown as jasplakinolide binds to F-actin at the same site as phalloidin thus preventing us from visualising F-actin in these cells [1]. Each image = 50 x 50 μm. (d) Confirmation that newly derived immortal dilute lethal 20j (d-l20j) derived melanocytes, melan-d1, lack myosin-Va protein. Lysates of melan-a and melan-d1 cells were immunoblotted with myosin-Va specific antibodies. Commassie blue (CB) staining confirm equal loading of lysate and also highlights a ~200kDa band present in melan-a, but not melan-d1 lysate that may correspond to myosin-Va (asterisk). (e) Flow cytometric analysis of F-actin levels in latrunculin A treated melan-a cells. melan-a cells were incubated for 1 hour with latrunculin A or solvent alone, trypsinised, fixed and stained with texas-red conjugated phalloidin. The fluorescence intensity of 10,000 cells within each population was then measured using a MoFlo fluorescence cytometer (as described in material and methods).

Figure S2 (Companion to main figure 2). Analysis of the intracellular distribution of GFP, microtubules and melanosomes in melan-d1 melanocytes using confocal immunofluorescence microscope. melan-d1 were incubated with 1μM nocodazole or DMSO 0.1% for 1 hour and then infected with adenovirus allowing expression of GFP-myosin-Va or GFP alone in the continued presence of the inhibitor/solvent. Cells were fixed 16 hours later and stained with GFP and α-tubulin specific antibodies (as decribed in Experimental procedures). The white squares in the merged images indicate the part of the cell that is presented in the high magnification panels below. Arrows in high magnification panels indicate examples of colocalisation between melanosomes and GFP-myosin-Va. N.B. in these images some melanosomes appear as hollow red 'O'-shaped structures this is likely to be due to shade-off and halo phase contrast artifacts. High magnification images show

myosin-Va is located adjacent to the highly refractive melanin core of melanosomes, this may reflect differences in z-plane between phase contrast and confocal images. Scale bar = 10μm.

Figure S3 (Companion to main figures 3 + 4). a) Analysis of the subcellular localisation of myosin proteins in melan-d1 melanocytes. i-iii) confocal images showing the intracellular distribution of GFP or the indicated myosin protein (green) and melanosomes (red in i) and ii) and phase/blue in iii)) and F-actin (red in iii)). i) and ii) correspond to proteins tested in main figures 3 and 4, respectively. Left-hand column are over-view images showing entire example cells, while right-hand column are high magnification images (dimensions 2.5 x 2.5μm) showing parts of these cells highlighted in the white boxes shown in the over-views. White circles (left-hand column) indicate the shape of the micropattern (diameter = 46μm). iii) highlights the association of the G440A mutant with F-actin in the peripheral cytoplasm of a free growing cell, lower row are high magnifications images of the boxed region in the upper row. Bar = 20μm. See Fig S2 legend for explanation of the difference in the distribution of GFP and melanin in high magnification images. b) The effect of mini-myosin and control proteins expression upon pigment distribution in melanocytes. i-iii) scatter-plots showing melanosome distribution in micropattern grown (i) or free-growing (ii-iii) melanocytes; melan-ln (Mlph deficient), melan-ash (Rab27a deficient) and melan-a. Melanosome distribution was analysed after either 24 (melan-ash and melan-a) or 48 hours (melan-ln) of plasmid transfection or virus infections, respectively. For ii and iii % pigment coverage (pigment filled area/total cell area) was calculated as described previously [2]. Horzontal bars indicate the median and 25th and 75th percentile of the population in each case. i-ii) The significance of differences in pigment distribution values (PDD or % pigment coverage) between populations are indicated above. iii) The significance of differences in % pigment coverage values for each population compared with the GFP and wild-type myosin-Va are displayed below and above each scatter (**, *** and **** indicate p=<0.01, p=<0.001 and p=<0.0001), respectively.

Figure S4 (companion to main figure 3). Acutely activatable myosin allows direct observation of myosin-Va driven organelle transport in living melanocytes. a) is a schematic summarising the design of the rapamycin (A/C heterodimeriser) activatable myosin-Va (see Supplementary experimental procedures for details). b) is a line-plot showing the change in melanosome distribution, as reported by pigment area, in melan-d1 melanocytes expressing the myosin-Va S1 and tail fragments before and after addition of A/C heterodimeriser (0.5 μM) in the presence and absence of microtubules (n=4 different cells in each case, error bars are standard error of the mean). Pigment area = the x,y area of cytoplasm filled by melanosomes at the indicated time divided by the equivalent area at t=0. c) selected confocal micrographs from one of the time-lapse sequences analysed in b. Upper and lower panel sets show images from sequence recorded from cells in the presence and absence of microtubules, respectively (bar = 20μm).

Experimental procedures.

Plasmid and virus constructs. pENTR529 GFPC2 myosin-Va containing the melanocyte specific exons D and F and lacking brain specific exon B of myosin-Va (XM_006510828.1) was generated by a four-step cloning procedure [3]. Myosin-Va fragments were amplified by RT-PCR from murine melanocytes. pENTR529 was generated from pENTR (Invitrogen) by insertion of EcoRI, NotI, KpnI, SacI and BamHI restriction sites. Step 1: myosin-Va a.a. 1258-1877 coding sequence was subcloned into pENTR529 using internal SacI and BamHI restriction sites to generate pENTR529 myosin-Va 1258-1877. Step 2: myosin-Va a.a 709-1258 coding sequence was subcloned into pENTR529 myosin-Va 1258-1877 using KpnI (internal) and SacI restriction sites to generate pENTR529 myosin-Va 709-1877. Step 3: EGFP coding sequence was PCR amplified from pEGFP-C2 (Clontech) and sub-cloned into pENTR529 myosin-Va 709-1828 using EcoRI/NotI restriction sites to generate pENTR529 GFPC2 myosin-Va 709-1877. Step 4: myosin-Va a.a 1-709 coding sequence subcloned into pENTR529 GFPC2 myosin-Va 709-1877 using NotI and KpnI restriction sites to generate pENTR529 GFPC2 myosin-Va. Myosin-Va mutants; G440A, S217A, 0IQ, 2IQ and 4IQ, (lacking amino acids 762-920, 810-920 and 858-920, respectively)were generated by Quikchange site-directed mutagenesis. Mini-myosin myosin-VI (+/-insert)/Va chimera (mini-VI+I, VI-I and Va) were as described in [4] and from the N-terminus comprised; EGFP fused to the motor domain of human myosin-VI (NM_010864.2) including (aa 1-809; VI+I) or lacking (aa 1-771; VI-I) the unique insert region responsible for the directionality of myosin-VI or murine myosin-Va (aa 1-761; Va), fused to the lever arm of murine myosin Va (aa 762-920) and the Rab27a binding domain of murine Sytl2a (aa 1-90; RBD) (NM_031394). For inducibly active myosin-Va 2 constructs were made; 1) Myosin-Va S1 (from the N-terminus) GFP-motor-lever-FKBP fusion was made by replacement of the RBD of the mini-Va construct with human FK506 binding protein 1A (FKBP) (NM_000801.4) (aa 1-108) (Source Bioscience) and 2) Myosin-Va tail (from the N-terminus) FRB-mCherry-myosin-Va (melanosome/melanophilin binding tail) was made by sequential sub-cloning of human FKBP-rapamycin–binding domain (FRB) of mTOR (mammalian target of Rapamycin; L35478.1) (aa34-127) (Source Bioscience), mCherry and murine myosin-Va (aa 1277-1895) into the pENTR-V5 vector previously described [2]. Primer sequences are available upon request. Adenoviruses allowing expression of the GFP-myosin-Va and mutant variants were generated as previously described [2].

Melanocyte culture. To generate immortal melan-d1 melanocytes C57BL6/J mice heterozygous for the dilute lethal-20J (d-l20J) mutation were crossed with C57BL6/J mice carrying an Ink4a-Arf exon 2 deletion [5]. Trunk skin of a neonatal F2 d-l20J/d-l20J mouse was used for preparation of melanocyte cultures, as described previously [6], yielding immortal line melan-d1. Confocal immunofluorescence microscopy and Western blotting confirmed that these cells contain a normal complement of mature melanosomes and lack expression of myosin-Va (Fig S1b+d). melan-d1 cells were maintained in RPMI 1640 medium supplemented with 10% fetal calf serum, 2 mM glutamine, 100 U/ml penicillin G, and 100 mg/ml streptomycin, 200 nM phorbol 12-myristate 13-acetate, 200 pM cholera toxin (all from Sigma-Aldrich, Poole, United Kingdom), at 37°C with 10% CO_2. Cultures of immortal melan-a, melan-ln and melan-ash cells were maintained as described previously [7]. Cytoskeleton inhibitors were added to full melanocyte growth medium to 1μM nocodazole, 25nM latrunculin-A and 8nM jasplakinolide.

Immunofluorescence microscopy. Cells for immunofluorescence were paraformaldehyde fixed, stained and fluorescence and transmitted light images of melanocytes were then collected using a

Zeiss LSM710 confocal microscope fitted with a 63x 1.4NA oil immersion Apochromat lens. All images presented are single sections in the *z*-plane. Antibodies and stains were used as indicated; mouse monoclonal anti-GFP (Roche 11814460001; 1:200) mouse-anti-α-tubulin (1:100 Sigma clone DM-1) texas-red-phalloidin (Sigma P1951; 100nM), goat anti-mouse Alexa488 labelled secondary antibodies (Molecular Probes A-11001; 1:500). For live cell experiments using activatable myosin-Va, melan-d1 cells were plated 35mm diameter glass bottomed petri dishes (Matek P35G-1.5-20-C) ($1x10^4$ cells/dish) and the next day infected with adenoviruses allowing expression of GFP-myosin-Va(S1)-FKBP and FRB-mCherry-myosin-Va tail. 48 hours later dishes were transferred to the stage of the Zeiss LSM710 confocal microscope within an environmental chamber (37°C), medium was replaced with L-15 medium supplemented with 10% fetal calf serum, 100 U/ml penicillin G, and 100 mg/ml streptomycin and 0.5μM rapamycin analogue (A/C heterodimeriser, Clontech). Images of GFP, mCherry and melanosomes were then acquired at a frame rate of 12/hour.

Immunoblotting. Immunoblotting was performed as described previously [7] using rabbit anti-myosin-Va (Cell Signaling Technology #3402 1:1000).

Flow cytometry. Samples were analysed using a Beckman Coulter MoFlo XDP flow cytometer, equipped with 488nm and 561nm lasers to obtain forward and side scatter and excite Texas-Red respectively. Emitted fluorescence light was collected using 615/20nm band pass filter. Data was analysed using Weasel Software Version 3.0.2 (The Walter and Eliza Hall Institute of Medical Research, Melbourne Australia). Cells were gated based upon based upon their forward and side scatter profile, to exclude debris and overall mean fluorescence intensity (MFI) were reported for each population.

References.

1. Bubb, M.R., Senderowicz, A.M., Sausville, E.A., Duncan, K.L., and Korn, E.D. (1994). Jasplakinolide, a cytotoxic natural product, induces actin polymerization and competitively inhibits the binding of phalloidin to F-actin. The Journal of biological chemistry *269*, 14869-14871.
2. Hume, A.N., Tarafder, A.K., Ramalho, J.S., Sviderskaya, E.V., and Seabra, M.C. (2006). A coiled-coil domain of melanophilin is essential for Myosin Va recruitment and melanosome transport in melanocytes. Molecular biology of the cell *17*, 4720-4735.
3. Seperack, P.K., Mercer, J.A., Strobel, M.C., Copeland, N.G., and Jenkins, N.A. (1995). Retroviral sequences located within an intron of the dilute gene alter dilute expression in a tissue-specific manner. EMBO J *14*, 2326-2332.
4. Park, H., Li, A., Chen, L.Q., Houdusse, A., Selvin, P.R., and Sweeney, H.L. (2007). The unique insert at the end of the myosin VI motor is the sole determinant of directionality. Proc Natl Acad Sci U S A *104*, 778-783.
5. Lavado, A., Matheu, A., Serrano, M., and Montoliu, L. (2005). A strategy to study tyrosinase transgenes in mouse melanocytes. BMC Cell Biol *6*, 18.
6. Sviderskaya, E.V., Hill, S.P., Evans-Whipp, T.J., Chin, L., Orlow, S.J., Easty, D.J., Cheong, S.C., Beach, D., DePinho, R.A., and Bennett, D.C. (2002). p16(Ink4a) in melanocyte senescence and differentiation. J Natl Cancer Inst *94*, 446-454.
7. Hume, A.N., Ushakov, D.S., Tarafder, A.K., Ferenczi, M.A., and Seabra, M.C. (2007). Rab27a and MyoVa are the primary Mlph interactors regulating melanosome transport in melanocytes. J Cell Sci *120*, 3111-3122.
